# Development and validation of a risk stratification tool for 28-day mortality based on D-dimer to platelet ratio in moderate-to -severe traumatic brain injury: a retrospective cohort study

**DOI:** 10.3389/fnins.2026.1908494

**Published:** 2026-08-11

**Authors:** Zhaoyi Wang, Ran Du, Yixin He, Pengwei Pan

**Affiliations:** Neurointensive Care Unit, The First Affiliated Hospital of Zhengzhou University, Zhengzhou, Henan, China

**Keywords:** coagulopathy, D-dimer to platelet ratio, early neurological deterioration, prediction model, traumatic brain injury

## Abstract

**Background:**

Moderate-to-severe traumatic brain injury (msTBI) is associated with significant mortality. Post-injury coagulopathy, involving elevated fibrinolysis (reflected by D-dimer) and platelet consumption (reflected by platelet count), contributes to acute neurological deterioration and poor outcomes. This study evaluated the potential association of the D-dimer to platelet ratio (DPR) with 28-day all-cause mortality and explored its link to early neurological progression as a mechanistic validation in patients with msTBI.

**Methods:**

This retrospective, single-center cohort study included 340 patients with msTBI. Patients were divided into training (*n* = 239) and split-sample validation (*n* = 101) groups. The primary endpoint was 28-day all-cause mortality. To enhance mechanistic alignment, an exploratory analysis was conducted using early neurological deterioration (END, defined as hemorrhagic progression, GCS decline, or pupillary abnormality within 72 h) as a proximal clinical outcome. A risk stratification tool was developed using Boruta feature selection and multivariable Cox proportional hazards regression. Exploratory logistic regression was applied to evaluate the association between admission DPR and END. Tool performance was evaluated via discrimination (Area Under the Curve, AUC), calibration, and decision curve analysis, with internal validation using bootstrap resampling and split-sample validation.

**Results:**

In the multivariable Cox regression, Glasgow Coma Scale (GCS), admission blood glucose (ABG), and DPR (per 1 SD increase) (HR: 1.558, 95% CI: 1.291–1.881, *P* < 0.001) were independently associated with 28-day mortality. In the exploratory END analysis, DPR was associated with acute progression (OR: 2.223, 95% CI: 1.367–3.614, *P* = 0.001), with an AUC of 0.724. The DPR-B risk stratification tool yielded a 28-day time-dependent AUC of 0.852 in the training group.

**Conclusion:**

Admission DPR is an independent risk marker associated with both acute neurological progression and 28-day mortality in patients with msTBI. The DPR-B tool, incorporating GCS, ABG, and DPR, offers a potential framework for early risk stratification; however, its clinical generalizability and translational value for guiding therapeutic interventions warrant further validation in prospective studies using more proximal, pathophysiologically relevant outcomes.

## 1 Introduction

Traumatic brain injury (TBI) remains a public health concern, contributing to a leading cause of death and disability globally among all trauma-related injuries despite advancements in medical care (Iaccarino et al., 2018). Worldwide, TBI accounted for 27.16 million incident cases, 48.99 million prevalent cases and 7.08 million years lived with disability (YLD) in 2019, according to epidemiological data (James et al., 2019). TBI can be clinically classified as mild, moderate or severe according to the Glasgow Coma Scale (GCS), and associated permanent disability rates are 10, 60, and 100%, respectively, with overall mortality rates of 20–30% (Rosenfeld et al., 2012). Moderate-to-severe TBI (msTBI), defined as GCS score of 3–12 on admission, carries a substantial burden of morbidity and mortality (Iaccarino et al., 2018). Large multicenter studies have shown that an estimated 10% of msTBI patients die within 6 months, and an additional 20% are fully dependent in all aspects of care (McCrea et al., 2021; Yue and Deng, 2023). Given this substantial disease burden, accurate risk stratification remains important. Recent registry data, such as the CENTER-TBI China study, highlight the challenge of cross-population generalizability in existing mortality prediction models for msTBI (Lang et al., 2023).

Identifying patients at high risk for mortality early is fundamental for effective risk stratification (Iaccarino et al., 2018). However, predicting clinical outcomes in patients with msTBI remains challenging due to the pathophysiological heterogeneity of the condition (Muehlschlegel et al., 2024). In recent years, several prognostic tools have been developed to address this, including clinical nomograms tailored for prehospital settings (Wang et al., 2024) or specific populations such as those requiring mechanical ventilation (Lu et al., 2024). Furthermore, systematic reviews of machine learning-based models have demonstrated favorable discriminative capacity (Wu et al., 2023). However, these tools often require specialized variables (e.g., detailed imaging findings, specific ventilator settings, or laboratory markers not universally available) or focus on restricted patient subgroups, which may limit their generalizability to the broader msTBI population (Eagle et al., 2023; Wu et al., 2023). Beyond these clinical-data-based approaches, current prognostic indicators still rely heavily on specialized biomarkers (e.g., S100β, GFAP, NSE, MBP) (Behzadi et al., 2024) that are often difficult to obtain in acute clinical settings. Consequently, there is a need to explore practical, integrated indicators that can assist in initial risk assessment at the bedside and in ICU settings.

Evidence from critical care medicine suggests that biomarkers derived from routine coagulation tests may hold prognostic value. Coagulopathy is a common and significant complication following msTBI, contributing to poor outcomes (Anderson et al., 2021; Chen et al., 2023; Hou et al., 2024). This condition stems from a disruption of the coagulation system, often leading to an imbalanced activation of both coagulation and fibrinolysis (Anderson et al., 2021; Chen et al., 2023). Hallmarks of this dysfunction are elevated fibrinolysis and impaired platelet function, which collectively drive the progression of intracranial hemorrhage and early neurological deterioration (END), subsequently increasing the risk of unfavorable outcomes (Anderson et al., 2021; Hou et al., 2024). D-dimer, a biomarker of fibrin degradation, rises rapidly after TBI, with peak levels around day 3 reflecting injury severity and serving as a predictor for hematoma expansion and unfavorable prognosis (Sugimoto et al., 2017; Chen et al., 2023; Zhao et al., 2023). The release of brain-derived, tissue factor-rich microvesicles is implicated in triggering this systemic coagulopathy and hyperfibrinolysis (Chen et al., 2023). Conversely, platelets are both consumed and functionally impaired post-TBI. Thrombocytopenia, particularly counts below 100×10^9^/L, is linked to mortality (Zhao et al., 2023; Hou et al., 2024). However, platelet count alone can be influenced by various confounders, limiting its standalone prognostic reliability (Zhao et al., 2023).

Given that D-dimer and platelet count represent distinct yet pathophysiologically linked aspects of post-TBI coagulopathy, their ratio (D-dimer to platelet ratio, DPR) may offer an integrated risk marker. This rationale is supported by findings in other critical illnesses where DPR has demonstrated predictive value. In sepsis, DPR has been identified as an independent predictor of 28-day mortality, demonstrating predictive efficacy (AUC = 0.939) (Zhao et al., 2023). Similarly, in cardiovascular emergencies, an elevated DPR is independently associated with in-hospital mortality in patients with acute Type A aortic dissection (Zhao et al., 2024). The prognostic value of DPR extends to infectious diseases and hepatology, where it predicts disease severity, intensive care unit admission, and mortality in COVID-19 patients (Tahmaz et al., 2023), and serves as a prognostic indicator comparable to established scoring systems in HBV-related decompensated cirrhosis (He and Ding, 2024).

Despite its established prognostic utility in various critical care settings, the specific role of DPR in predicting outcomes, particularly 28-day mortality, in patients with msTBI remains unexplored. Compared with existing prognostic models that may require high-dimensional data or complex computational algorithms (Eagle et al., 2023; Wu et al., 2023), there is a need for simplified tools that balance clinical accessibility with pathophysiological interpretability. Therefore, the objective of our study was to evaluate the association of DPR with 28-day mortality in msTBI patients, to develop a risk stratification tool incorporating DPR, and to explore its link to END for mechanistic validation. This study is primarily intended as an exploratory, hypothesis-generating analysis to determine whether a DPR-based tool could provide a preliminary framework for risk stratification, identifying signals that warrant further validation in future multicenter prospective studies (Warman et al., 2022; Eagle et al., 2023).

## 2 Materials and methods

### 2.1 Study design and patients

**FIGURE 1:**
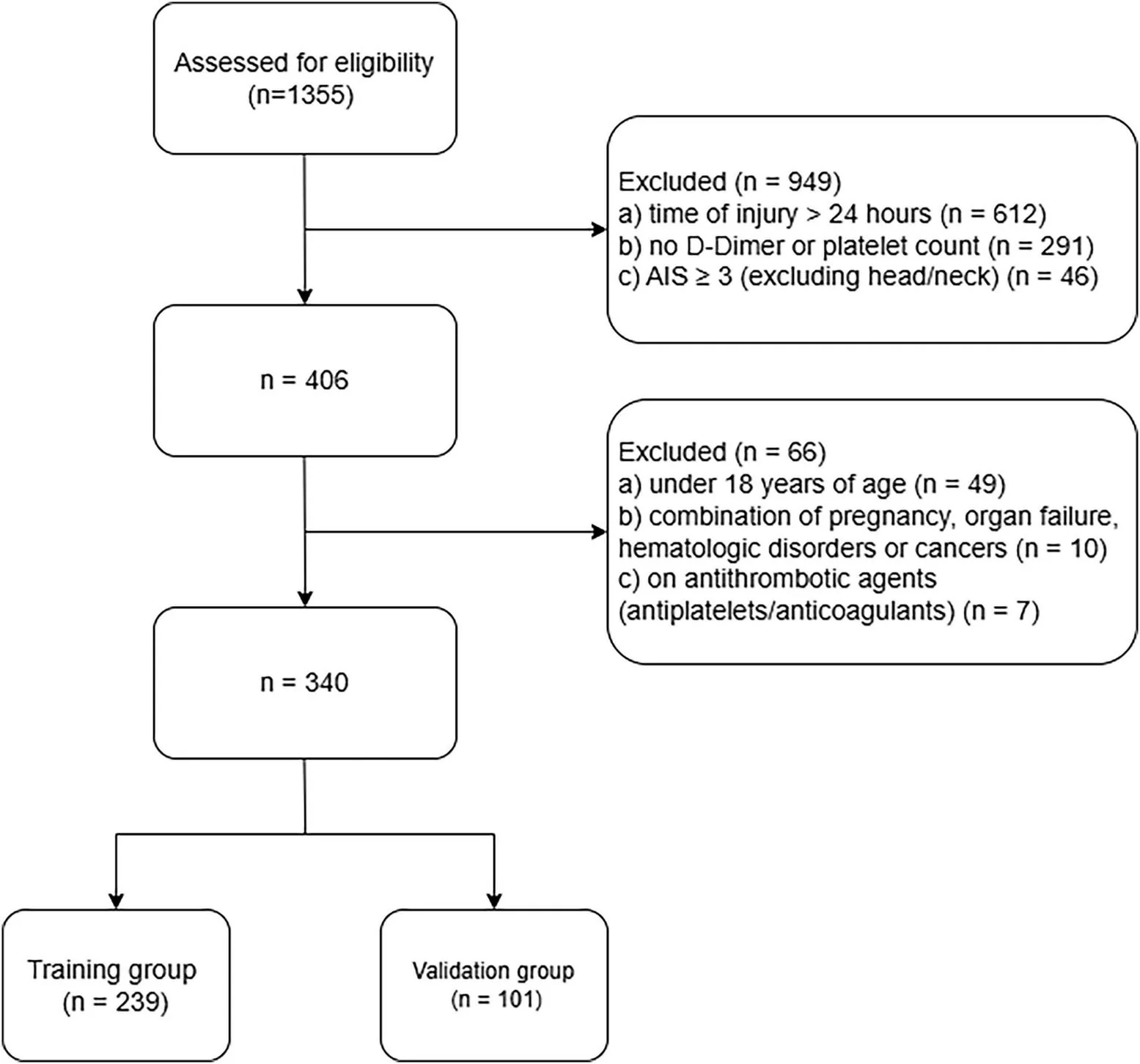
Flowchart.

This retrospective cohort study involved patients with msTBI who were admitted to the intensive care unit (ICU) of The First Affiliated Hospital of Zhengzhou University between January 1, 2020, and August 31, 2024. Patients were divided into a training group and a split-sample validation group in a 7:3 ratio using stratified sampling, with a fixed random seed (123) to ensure reproducibility. The training group was employed to identify independent prognostic factors for short-term mortality and to develop a new risk stratification tool. The split-sample validation group was then used to evaluate the performance of this tool. Inclusion criteria: (1) Age ≥ 18 years; (2) Clear history of trauma with injury onset ≤ 24 h before admission; (3) Brain CT scan performed within 24 h after admission; (4) Glasgow Coma Scale (GCS) score 3–12 at admission: severe (GCS score 3–8), moderate (GCS score 9–12). Exclusion Criteria: (1) Missing D-Dimer or platelet count data within 24 h of admission; (2) Abbreviated Injury Scale (AIS) score ≥ 3 for injuries outside the head/neck region; (3) Comorbid conditions including pregnancy, heart failure, renal failure, malignancies, or hematologic disorders; (4) Current use of anticoagulant or antiplatelet medications (Figure 1).

### 2.2 Clinical data

Demographic and clinical data were retrospectively extracted from records in the inpatient information management system. Baseline laboratory variables for all subjects were measured within 24 h of admission. Demographic variables included gender and age. Clinical variables included the following: (1) Initial vital signs recorded upon ICU admission: temperature, heart rate (HR), respiratory rate (RR), and mean arterial pressure (MAP); (2) Initial laboratory tests recorded upon ICU admission: admission blood glucose (ABG), absolute counts of white blood cells, neutrophils and lymphocytes, hemoglobin (Hb), platelet counts (PLT), potassium, sodium, chloride, calcium, urea, creatinine, alanine aminotransferase (ALT), aspartate aminotransferase (AST), gamma-glutamyl transferase (GGT), albumin (ALB), total bilirubin (TBIL), lactate, international normalized ratio (INR), and D-dimer; (3) Neurological evaluation: first recorded GCS score and pupillary size on admission; (4) Others: time of injury, history of hypertension, and previous diabetes treatment.

To support the mechanistic validation of DPR, follow-up neurological and imaging data within 72 h of admission were also collected, including subsequent GCS scores, changes in pupillary light reflex or diameter, and follow-up brain CT scans to assess hemorrhagic progression.

### 2.3 Definitions

The time of injury was defined as the duration between trauma occurrence and ICU admission. Hypertension diagnosis adhered to the 2018 ESC/ESH Guidelines for the Management of Arterial Hypertension (Williams et al., 2018). Neurological evaluation comprised GCS score and pupillary size, independently assessed by ≥ 2 ICU physicians, with asymmetry of pupillary defined as ≥ 0.5 mm interpupillary diameter discrepancy. Neurosurgical interventions were classified as decompressive procedures (craniectomy/craniotomy) and/or hematoma evacuation (intracranial, epidural, or subdural), including ventricular drainage when clinically indicated.

### 2.4 Outcomes

Patient survival was assessed from admission through day 28 by medical record review and telephone follow-up. The primary outcome was 28-day all-cause mortality. Survival time was defined as the interval from hospital admission to either the occurrence of death or the date of the last known clinical contact.

Early neurological deterioration (END) within 72 h of admission was assessed as a proximal endpoint to validate the mechanistic relevance of DPR. END was defined as the occurrence of any of the following within 72 h of admission (Vásquez-García et al., 2025): (1) Hemorrhagic progression on follow-up head CT: for measurable parenchymal hematomas, an increase in volume ≥33% or ≥6 mL; for non-measurable or multi-compartment hemorrhage (e.g., subarachnoid, subdural, epidural, intraventricular), new hemorrhage or unequivocal increase in hemorrhagic burden on follow-up CT, as determined by the original radiology report and confirmed by a reviewing neurologist; (2) A decline in Glasgow Coma Scale (GCS) score: ≥2-point decrease in total score or ≥1-point decrease in the motor component; (3) Newly developed pupillary abnormality: interpupillary asymmetry ≥0.5 mm or abnormal light reflex compared with admission; (4) Death within 72 h of admission. Patients who did not undergo repeat CT and had no clinical deterioration (no GCS decline, no pupillary abnormality, and did not die within 72 h) were excluded from the END analysis.

### 2.5 Exposure assessment

The primary exposure variable, DPR, was calculated by collecting admission D-dimer levels and platelet counts, using the formula: DPR = D-dimer level (mg/L) ×1,000/platelet count level (×10^9^/L). Given the right-skewed distribution of DPR and D-dimer in the training group (see Supplementary Figures S1, S2), raw, log-transformed, and categorized forms were evaluated. Linearity of the relationship with the log-hazard was assessed using restricted cubic splines (RCS), and model fit was compared using the Akaike Information Criterion (AIC). DPR was selected as the primary predictor based on these comparisons. For multivariable analysis, DPR was standardized (per 1 standard deviation increase). Sensitivity analysis using winsorization at the 95th percentile was conducted to assess the influence of extreme values on model robustness. Before investigating the association between DPR values and the risk of 28-day all-cause mortality, the Boruta machine learning algorithm was utilized for feature selection to assess the significance of each variable in the analytical model (Degenhardt et al., 2019). For multivariable analysis, DPR was standardized (per 1 standard deviation increase) to ensure comparability of regression coefficients. The nomogram, however, uses raw DPR values to facilitate clinical application.

### 2.6 Statistical analysis

Model development and reporting followed the TRIPOD guidelines to ensure transparency. Continuous variables are presented as medians with interquartile ranges (IQR). Categorical variables are summarized as frequencies with percentages (%). Group comparisons were performed using the Wilcoxon rank-sum test for continuous variables and the Chi-square test for categorical variables. Given the time-to-event nature of the data, the Cox proportional hazards regression model was employed as the primary analytical tool for predicting 28-day mortality. A detailed summary of missing values is provided in Supplementary Table S1. For the 21 patients lost to follow-up, data were handled as right-censored observations at the time of their last clinical contact. Patients surviving beyond the 28-day period were administratively censored at day 28. The study cohort (*n* = 340) was randomly partitioned into a training group (*n* = 239) and a split-sample validation group (*n* = 101) using a 7:3 ratio. The sample size was justified based on the Events Per Variable (EPV) criteria. In the training set, there were 57 death events; for the final model involving three independent predictors, the EPV was 19.0, exceeding the threshold of 10 required to maintain model performance and reduce the risk of overfitting. Feature selection was conducted using the Boruta machine learning algorithm (500 runs) to identify variables with confirmed importance. Compared to linear shrinkage methods like LASSO, Boruta is a random forest-based wrapper that excels in capturing high-dimensional interactions and adheres to the “all-relevant” selection principle, ensuring potential prognostic factors are not prematurely excluded. Variables identified by Boruta were further screened using univariable Cox regression. Significant variables from univariable Cox regression (*P* < 0.05) were included in a multivariable model, following an assessment of multicollinearity using Variance Inflation Factors (VIF) and a Spearman correlation matrix (Supplementary Figure S3). The Schoenfeld residuals were used to verify the proportional hazards (PH) assumption. Model performance was evaluated via the Concordance index (C-index), 28-day time-dependent Area Under the Curve (AUC), and the Brier Score. Calibration curves (28-day) were utilized to assess model calibration.

As an exploratory analysis to ensure mechanistic alignment, the association between DPR and END was evaluated in the training cohort. Given the binary nature of END within a fixed 72-h window, multivariable logistic regression was employed to identify independent predictors of END. This analysis was performed in the training group only, given its exploratory nature. Candidate variables were selected using the Boruta algorithm (500 runs), consistent with the primary analysis. Variables with variance inflation factor < 5 were entered into the multivariable model. The discriminative ability of DPR alone for predicting END was assessed using the area under the receiver operating characteristic curve (AUC). Patients without follow-up CT and without clinical deterioration (no GCS decline, no pupillary abnormality, and did not die within 72 h) were excluded from this analysis, as described in section 2.4.

Decision curve analysis (DCA) was performed to assess the potential net benefit of the DPR-B risk stratification tool across a range of threshold probabilities, based on the assumption that identifying high-risk patients might prompt closer monitoring (Vickers and Elkin, 2006). The Target Model yielded positive net benefit across the threshold probability range in both the training and validation cohorts and exceeded the Baseline model. In the validation cohort at a threshold probability of 0.2, the Target Model achieved higher net benefit than the Full Model (Difference: 0.0204; 95% CI: 0.0076–0.0354; *P* < 0.001). The Full Model included more variables but showed lower net benefit in the validation cohort, which suggests potential overfitting. The Target Model maintained higher net benefit with fewer variables. Internal validation was performed using bootstrap resampling in the training group and split-sample validation in the validation group. Kaplan-Meier survival curves with log-rank tests were generated based on the optimal DPR cut-off determined by the Youden index. Static and dynamic nomograms were developed to visualize the model.

To assess the robustness of our findings, a multivariable logistic regression analysis was conducted as a sensitivity analysis using the same candidate variables. This was performed to assess the consistency of the identified prognostic factors for the binary outcome of 28-day mortality. All analyses were performed using R software (version 4.5.1) with packages including survival, rms, Boruta, timeROC, pROC, riskRegression, survminer, ggplot2, patchwork, gtsummary, flextable, broom, car, shiny, shinydashboard, and plotly.

### 2.7 Ethics approval and consent to participate

This study was conducted with the approval of the Ethics Committee of The First Affiliated Hospital of Zhengzhou University (Approval No. 2025-KY-1846) and in compliance with the Declaration of Helsinki. The requirement for obtaining informed consent was waived by the Ethics Committee due to the retrospective nature of this research.

## 3 Results

### 3.1 Study population

This study included a total of 340 patients, and the study flowchart is presented in Figure 1. As shown in Table 1, baseline characteristics were generally well-balanced between the training group (*n* = 239) and the split-sample validation group (*n* = 101). No statistically significant differences were observed for the majority of variables (all *P* > 0.05), with the exception of platelet count (*P* = 0.022), creatinine (*P* = 0.032), albumin (*P* = 0.019), and INR (*P* = 0.022), indicating a generally well-balanced cohort division for modeling. In the training group (73.22% male), the median age was 53 years, and the 28-day all-cause mortality and END rates were 23.85 and 24.89%, respectively.

**TABLE 1:** Baseline characteristics comparison between training and validation groups.

Variable	Training group (*n* = 239)	Validation group (*n* = 101)	*P*-value
Demographics			
Age, years	53.00 (39.00–63.00)	56.00 (44.00–65.00)	0.263
Gender, n (%)			0.154
Male	175 (73.22)	82 (81.19)	
Female	64 (26.78)	19 (18.81)	
Vital signs			
Temperature, °C	36.80 (36.50–37.30)	36.90 (36.60–37.40)	0.219
HR, bpm	90 (77–108)	87 (76–108)	0.712
RR, bpm	20 (16–22)	20 (16–23)	0.728
MAP, mmHg	100.00 (85.67–111.00)	100.67 (87.33–110.00)	0.998
Laboratory data			
WBC, × 10^9^/L	15.01 (11.95–18.20)	13.96 (10.87–18.43)	0.269
NEUT, × 10^9^/L	13.09 (10.30–16.01)	12.04 (9.08–15.95)	0.18
LYM, × 10^9^/L	0.75 (0.48–1.23)	0.82 (0.48–1.39)	0.593
Hb, g/L	127.00 (110.00–138.00)	119.00 (102.00–140.00)	0.115
PLT, × 10^9^/L	190.00 (145.00–238.00)	166.00 (124.00–207.00)	0.022
ABG, mmol/L	9.20 (7.65–11.30)	9.60 (7.50–12.60)	0.565
Potassium, mmol/L	3.62 (3.31–3.89)	3.49 (3.29–3.81)	0.119
Sodium, mmol/L	139.10 (137.00–142.00)	139.40 (136.00–142.00)	0.669
Chloride, mmol/L	103.15 (101.00–107.00)	105.00 (101.00–108.30)	0.139
Calcium, mmol/L	2.12 (2.02–2.22)	2.10 (1.97–2.22)	0.216
Urea, mmol/L	5.06 (4.00–6.46)	5.50 (4.20–6.90)	0.135
Creatinine, μmol/L	61.00 (49.60–77.00)	66.00 (55.00–79.00)	0.032
ALT, U/L	23.00 (16.00–41.00)	22.00 (15.00–34.00)	0.392
AST, U/L	39.00 (27.00–64.00)	38.00 (27.00–58.00)	0.906
GGT, U/L	24.00 (15.00–44.00)	21.00 (15.00–38.00)	0.154
ALB, g/L	40.15 (37.15–43.60)	39.00 (34.50–42.30)	0.019
TBIL, μmol/L	12.60 (9.20–17.50)	13.00 (8.20–19.10)	0.879
Lactate, mmol/L	2.40 (1.50–4.20)	2.16 (1.20–3.80)	0.384
INR	1.09 (1.00–1.19)	1.14 (1.05–1.24)	0.022
D-dimer, mg/L	6.57 (2.30–15.27)	6.10 (2.76–22.06)	0.687
DPR	40.68 (11.81–89.77)	41.04 (14.09–135.71)	0.323
Neurological evaluation			
GCS	5 (3–8)	5 (3–8)	0.443
Pupillary size, n (%)			0.156
Symmetry	152 (63.60)	73 (72.28)	
Asymmetry	87 (36.40)	28 (27.72)	
Complication			
Hypertension, n (%)			0.67
No	198 (82.85)	81 (80.20)	
Yes	41 (17.15)	20 (19.80)	
Diabetes, n (%)			0.145
No	216 (90.38)	85 (84.16)	
Yes	23 (9.62)	16 (15.84)	
Time of injury, hour	8.00 (3.00–16.00)	10.00 (4.00–20.00)	0.111
Outcomes			
Survival time, day	28 (10–28)	28 (11–28)	0.919
28-day ACM, n (%)			> 0.999
non-death	182 (76.15)	77 (76.24)	
Death	57 (23.85)	24 (23.76)	
END, n (%)	58 (24.89)	21 (21.21)	0.562

HR, Heart rate; RR, respiratory rate; MAP, mean arterial pressure; WBC, White Blood Cell; NEUT, neutrophil; LYM, lymphocyte; Hb, hemoglobin; PLT, platelet; ABG, admission blood glucose; ALT, alanine aminotransferase; AST, aspartate aminotransferase; GGT, gamma-glutamyl transferase; ALB, albumin; TBIL, total bilirubin; INR, international normalized ratio; DPR, D-dimer to Platelet Ratio; GCS, Glasgow Coma Scale; 28-day ACM, 28-day all-cause mortality; END, early neurological deterioration.

### 3.2 Baseline comparison between the death and non-death groups in the training group

Baseline characteristics of the training group, stratified by survival status at 28 days, are presented in Table 2. Compared with the non-death group, patients in the death group exhibited significant differences in vital signs, including a higher heart rate (*P* = 0.003) but lower temperature (*P* = 0.007), respiratory rate (*P* = 0.011), and mean arterial pressure (*P* = 0.038). Regarding laboratory data, the death group had significantly higher levels of INR, D-dimer, DPR, ABG, lactate, urea, creatinine, and aspartate aminotransferase (all *P* < 0.05). Conversely, significantly lower levels of calcium (*P* = 0.009), albumin (*P* = 0.003), and platelet count (*P* = 0.003) were observed in the death group. In terms of neurological evaluation, the GCS score was significantly lower in the death group (*P* < 0.001). Additionally, a history of previous diabetes treatment was more prevalent among patients in the death group (*P* = 0.039). Finally, the incidence of END was significantly higher in the death group than in the non-death group (73.08% vs. 11.05%, *P* < 0.001).

**TABLE 2:** Baseline comparison between the non-death and death groups (training group).

Variable	Non-death group (*n* = 182)	Death group (*n* = 57)	*P*-value
Demographics			
Age, years	53 (38–62)	56 (45–66)	0.256
Gender, n (%)			> 0.999
Male	133 (73.08)	42 (73.68)	
Female	49 (26.92)	15 (26.32)	
Vital signs			
Temperature, °C	36.8 (36.6–37.3)	36.7 (36.3–37.0)	0.007
HR, bpm	88.5 (74.0–103.0)	100.0 (80.0–120.0)	0.003
RR, bpm	20 (17–23)	18 (15–21)	0.011
MAP, mmHg	101.7 (86.7–111.0)	93.7 (78.3–109.7)	0.038
Laboratory data			
WBC, × 10^9^/L	14.87 (11.95–18.00)	15.42 (12.06–18.20)	0.762
NEUT, × 10^9^/L	13.03 (10.71–16.01)	13.62 (9.40–15.98)	0.857
LYM, × 10^9^/L	0.74 (0.47–1.18)	0.84 (0.51–1.85)	0.138
Hb, g/L	128.4 (113.0–138.0)	122.0 (103.0–136.0)	0.116
PLT, × 10^9^/L	197.5 (158.0–241.0)	164.0 (111.0–209.0)	0.003
ABG, mmol/L	8.8 (7.5–10.7)	11.3 (8.4–14.7)	< 0.001
Potassium, mmol/L	3.63 (3.35–3.88)	3.54 (3.13–3.91)	0.174
Sodium, mmol/L	139.0 (137.0–142.0)	140.0 (137.0–143.5)	0.613
Chloride, mmol/L	103.3 (101.0–107.0)	103.0 (100.0–107.0)	0.975
Calcium, mmol/L	2.14 (2.04–2.24)	2.09 (1.97–2.17)	0.009
Urea, mmol/L	4.9 (4.0–6.2)	5.5 (4.4–7.2)	0.029
Creatinine, μmol/L	60 (48–70)	75 (54–95)	0.002
ALT, U/L	22 (15–42)	26 (19–36)	0.383
AST, U/L	35 (26–58)	55 (36–73)	< 0.001
GGT, U/L	22.5 (15.0–45.0)	27.0 (16.0–35.0)	0.596
ALB, g/L	40.8 (37.8–43.8)	38.3 (33.3–42.0)	0.003
TBIL, μmol/L	12.8 (9.2–17.4)	12.1 (9.8–17.7)	0.785
Lactate, mmol/L	2.0 (1.4–3.3)	3.2 (2.1–5.1)	< 0.001
INR	1.07 (0.99–1.15)	1.19 (1.05–1.30)	< 0.001
D-dimer, mg/L	5.51 (2.06–12.29)	13.21 (5.14–47.79)	< 0.001
DPR	27.86 (9.43–76.30)	90.73 (32.62–351.15)	< 0.001
Neurological evaluation			
GCS	6 (4–9)	3 (3–4)	< 0.001
Pupillary size, n (%)			0.694
Symmetry	114 (62.64)	38 (66.67)	
Asymmetry	68 (37.36)	19 (33.33)	
Complication			
Hypertension, n (%)			> 0.999
No	151 (82.97)	47 (82.46)	
Yes	31 (17.03)	10 (17.54)	
Diabetes, n (%)			0.039
No	169 (92.86)	47 (82.46)	
Yes	13 (7.14)	10 (17.54)	
Time of injury, hour	8.25 (3.00–16.00)	7.00 (4.00–12.00)	0.478
Outcomes			
Survival time, day	28 (28–28)	4 (2–7)	< 0.001
END, n (%)	20 (11.05)	38 (73.08)	< 0.001

HR, Heart rate; RR, respiratory rate; MAP, mean arterial pressure; WBC, White Blood Cell; NEUT, neutrophil; LYM, lymphocyte; Hb, hemoglobin; PLT, platelet; ABG, admission blood glucose; ALT, alanine aminotransferase; AST, aspartate aminotransferase; GGT, gamma-glutamyl transferase; ALB, albumin; TBIL, total bilirubin; INR, international normalized ratio; DPR, D-dimer to Platelet Ratio; GCS, Glasgow Coma Scale; END, early neurological deterioration.

### 3.3 Identification of independent prognostic factors for 28-day all-cause mortality

**FIGURE 2:**
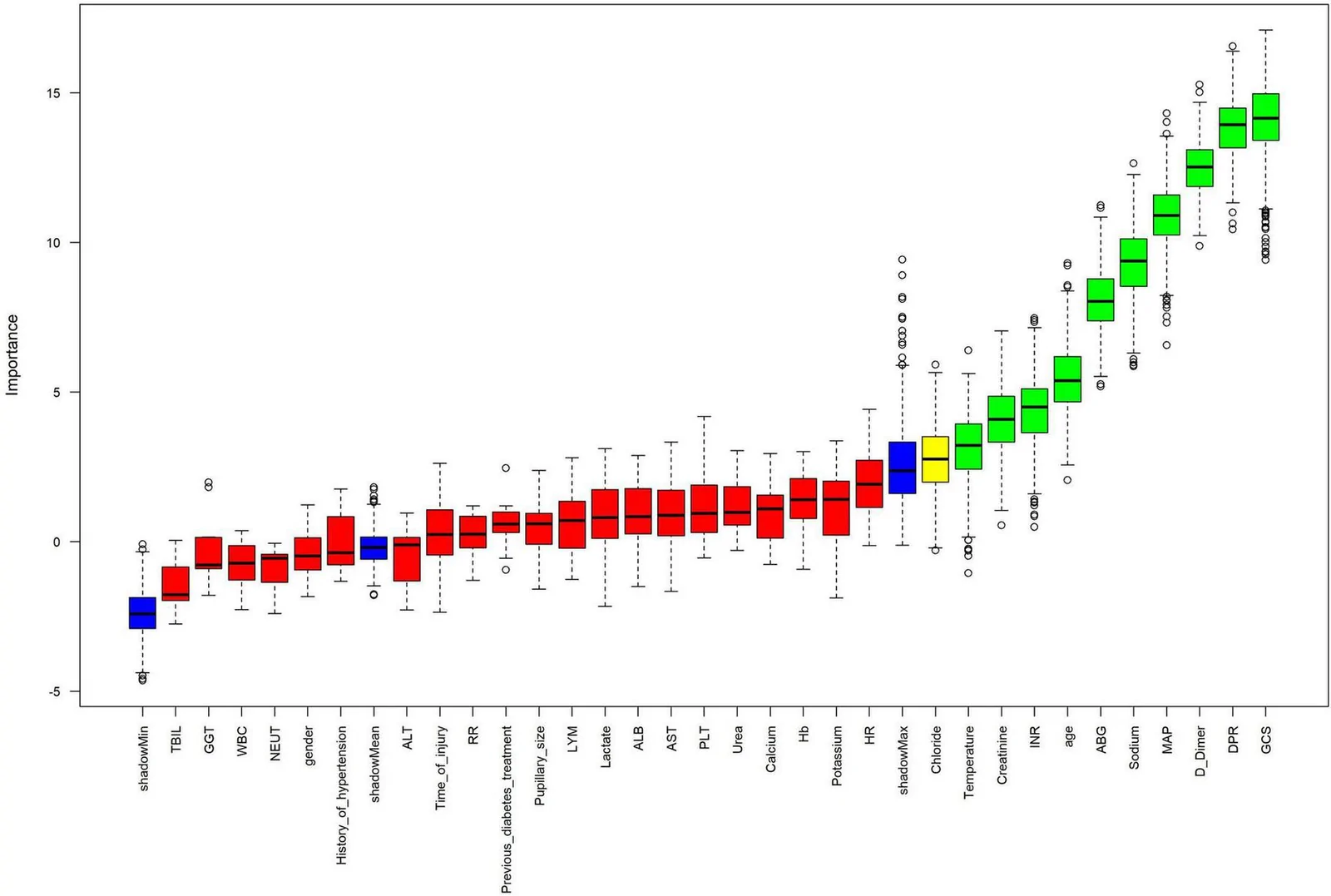
Feature importance ranking determined by the Boruta algorithm. HR, heart rate; RR, respiratory rate; MAP, mean arterial pressure; WBC, White Blood Cell; NEUT, neutrophil; LYM, lymphocyte; Hb, hemoglobin; PLT, platelet; ABG, admission blood glucose; ALT, alanine aminotransferase; AST, aspartate aminotransferase; GGT, gamma-glutamyl transferase; ALB, albumin; TBIL, total bilirubin; INR, international normalized ratio; DPR, D-dimer to Platelet Ratio; GCS, Glasgow Coma Scale.

Using the Boruta machine learning algorithm, 10 variables were selected as relevant features for 28-day all-cause mortality: DPR, GCS, D-dimer, MAP, sodium, ABG, age, INR, creatinine, and temperature (Figure 2). Multicollinearity among the variables was assessed using the variance inflation factor (VIF) test, and highly correlated variables were excluded. Based on univariable Cox proportional hazards analysis (Table 3), variables with *P* < 0.05 or established clinical relevance were entered into the multivariable Cox model. These included temperature, MAP, GCS, ABG, sodium, INR, and DPR. To determine the optimal functional form of DPR for the multivariable model, we compared raw, log-transformed, and categorical forms within a multivariable Cox regression framework. Linearity assessment using RCS and model fit comparison via AIC indicated that raw DPR yielded the lowest AIC (533.20) and displayed a linear relationship with the log-hazard; raw DPR was therefore selected for subsequent analyses (Supplementary Figures S4, S5 and Supplementary Table S2). Sensitivity analysis using 95th percentile winsorization showed a change in the hazard ratio from 1.522 to 1.449 (relative change: 4.80%) (Supplementary Table S3). Accordingly, we incorporated DPR into the final multivariable Cox regression model. Multivariable analysis demonstrated that GCS (HR: 0.705, 95% CI: 0.596–0.835, *P* < 0.001), ABG (HR: 1.068, 95% CI: 1.002–1.139, *P* = 0.044), and DPR (per 1 SD increase) (HR: 1.558, 95% CI: 1.291–1.881, *P* < 0.001) were independent prognostic factors for 28-day all-cause mortality (Table 3). The model satisfied the proportional hazards assumption, with a global *P*-value of 0.31 (Supplementary Table S4). Furthermore, the multivariable logistic regression analysis yielded results consistent with the primary Cox proportional hazards model. GCS, ABG, and DPR—remained independent predictors of 28-day mortality (all *P* < 0.05), supporting the consistency of our findings (Supplementary Table S5).

**TABLE 3:** Univariate and multivariate Cox proportional hazards analysis of 28-day all-cause mortality in msTBI.

Variable	Univariate analysis HR (95% CI)	Univariate analysis *P*-value	Multivariate analysis HR (95% CI)	Multivariate analysis *P*-value
Temperature	0.539 (0.345–0.841)	0.003	0.842 (0.594–1.195)	0.337
MAP	0.982 (0.968–0.997)	0.015	0.996 (0.983–1.010)	0.597
GCS	0.625 (0.526–0.742)	< 0.001	0.705 (0.596–0.835)	< 0.001
ABG	1.161 (1.103–1.223)	< 0.001	1.068 (1.002–1.139)	0.044
Sodium	1.071 (1.016–1.129)	0.017	1.040 (0.989–1.094)	0.13
INR	5.036 (2.639–9.613)	< 0.001	1.563 (0.608–4.021)	0.354
DPR (per 1 SD)	1.902 (1.642–2.204)	< 0.001	1.558 (1.291–1.881)	< 0.001
Age	1.012 (0.995–1.030)	0.164	–	–
Creatinine	1.003 (0.999–1.006)	0.166	–	–
D-Dimer (per 1 SD)	1.961 (1.627–2.364)	< 0.001	–	–

DPR and D-Dimer were standardized (per 1 SD increase) in the Cox regression. The nomogram uses raw DPR values for clinical calculation. HR, hazard ratio; CI, confidence interval; MAP, mean arterial pressure; GCS, Glasgow Coma Scale; ABG, admission blood glucose; INR, international normalized ratio; DPR, D-dimer to platelet ratio.

### 3.4 DPR risk stratification and its association with 28-day mortality

**FIGURE 3:**
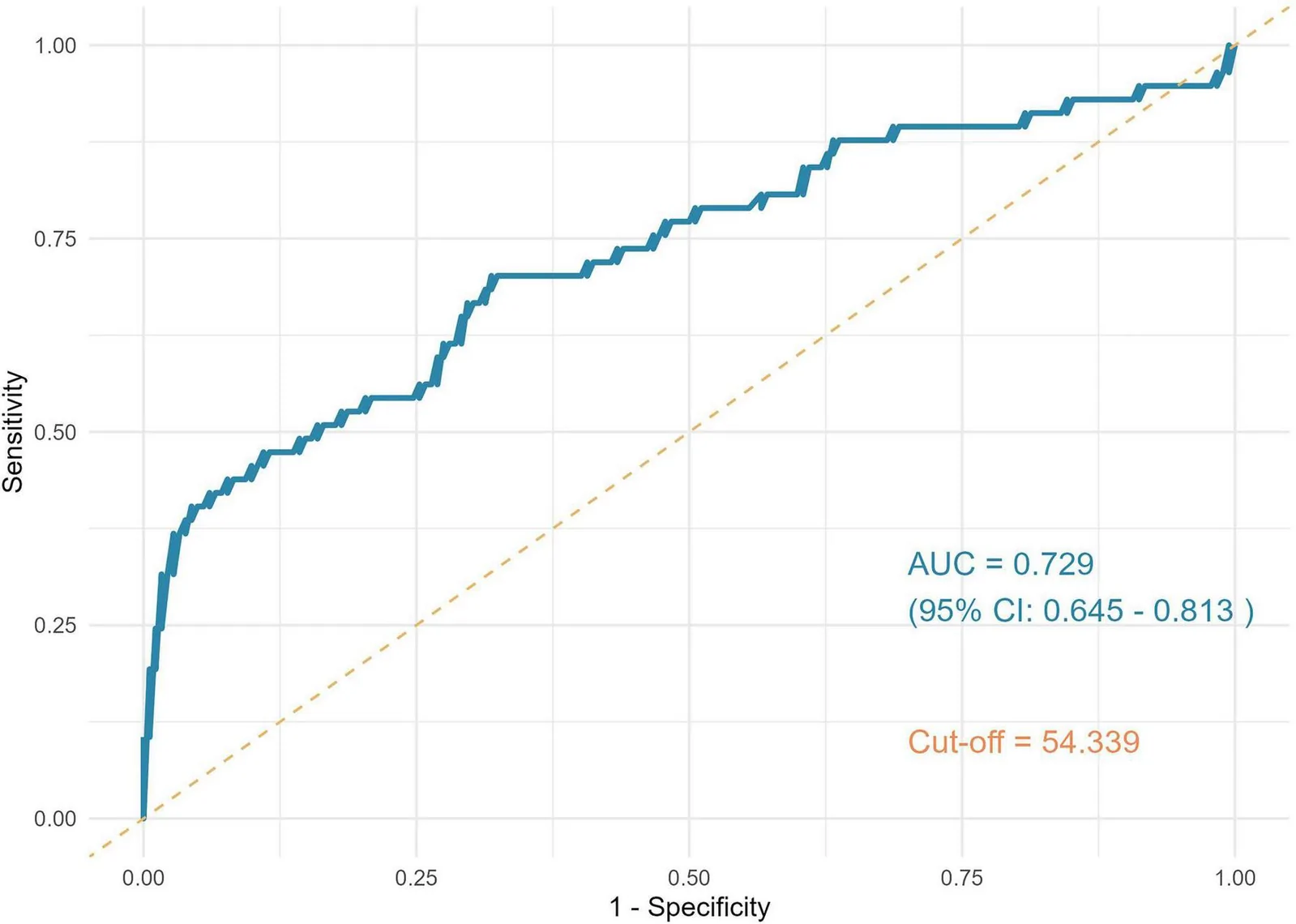
Receiver operating characteristic (ROC) curve of DPR in discriminating the risk of 28-day mortality. The AUC of 0.729 (95% CI: 0.645–0.813) reflects moderate discriminative performance in distinguishing mortality risk among patients with msTBI. The optimal cut-off of 54.339 serves as a potential indicator of early hemostatic imbalances, which may help identify patients at higher risk of clinical progression.

**FIGURE 4:**
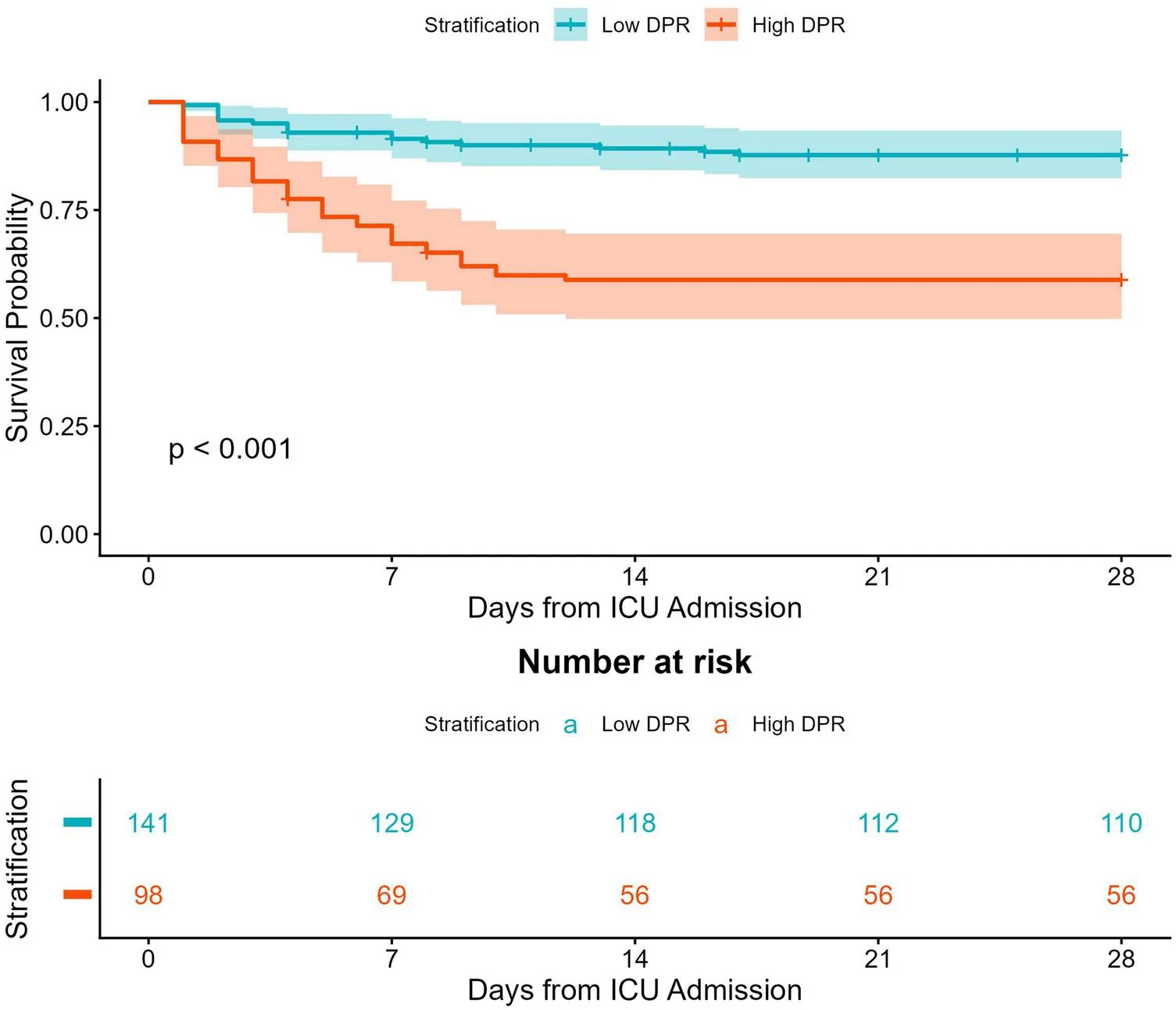
Kaplan-Meier survival curves for 28-day all-cause mortality stratified by DPR. Patients in the high DPR group ( ≥ 54.339) exhibited a lower survival probability compared to those in the low group ( < 54.339). The shaded areas represent 95% confidence intervals. Log-rank test, P < 0.001.

Multivariable Cox regression analysis indicated DPR as a risk factor for 28-day all-cause mortality in patients with msTBI. The area under the receiver operating characteristic curve (AUC) for discriminating 28-day mortality was 0.729 (95% CI: 0.645–0.813) (Figure 3). Patients were stratified into high (≥54.339, *n* = 98) and low (<54.339, *n* = 141) DPR groups based on the cut-off value determined by the Youden index. In the training group, this threshold yielded a sensitivity of 70.2% and a specificity of 68.1%. To confirm its stability, this pre-specified cut-off was applied to the validation group, demonstrating a sensitivity of 83.3% and a specificity of 66.2% (detailed in Supplementary Table S6). The 28-day mortality rate was 40.82% in the high DPR group and 12.06% in the low DPR group (*P* < 0.001, Table 4). Kaplan-Meier survival analysis demonstrated a difference in survival probability between the two groups (Log-rank test, *P* < 0.001; Figure 4).

**TABLE 4:** Comparison of baseline characteristics between the high and low DPR group (training group).

Variable	Low DPR group (*n* = 141)	High DPR group (*n* = 98)	*P*-value
Demographics			
Age, years	52.00 (38.00–59.00)	56.00 (45.00–66.00)	0.047
Gender, n (%)			0.825
Male	102 (72.34)	73 (74.49)	
Female	39 (27.66)	25 (25.51)	
Vital signs			
Temperature, °C	36.80 (36.70–37.30)	36.70 (36.50–37.00)	0.005
HR, bpm	90.00 (77.00–106.00)	89.00 (77.00–111.00)	0.828
RR, bpm	20.00 (18.00–22.00)	20.00 (15.00–22.00)	0.078
MAP, mmHg	100.00 (87.33–110.67)	96.67 (81.67–111.67)	0.233
Laboratory data			
WBC, × 10^9^/L	14.78 (11.99–17.00)	15.57 (11.07–18.80)	0.42
NEUT, × 10^9^/L	12.86 (10.77–15.36)	13.87 (9.70–16.80)	0.389
LYM, × 10^9^/L	0.80 (0.48–1.20)	0.68 (0.48–1.30)	0.797
Hb, g/L	129.00 (112.00–139.00)	123.50 (107.00–138.00)	0.272
PLT, × 10^9^/L	201.00 (173.00–255.00)	160.00 (101.00–208.00)	< 0.001
ABG, mmol/L	8.50 (7.20–10.60)	10.50 (8.40–12.73)	< 0.001
Potassium, mmol/L	3.65 (3.38–3.87)	3.54 (3.21–3.96)	0.471
Sodium, mmol/L	139.50 (137.00–142.00)	139.00 (137.00–142.20)	0.789
Chloride, mmol/L	104.00 (101.20–107.00)	103.00 (100.00–107.00)	0.248
Calcium, mmol/L	2.14 (2.04–2.24)	2.11 (2.01–2.20)	0.099
Urea, mmol/L	5.00 (3.90–6.20)	5.20 (4.30–6.70)	0.133
Creatinine, μmol/L	61.00 (51.00–72.00)	61.50 (47.00–80.00)	0.64
ALT, U/L	21.00 (14.00–36.00)	28.00 (19.00–48.00)	0.004
AST, U/L	32.00 (23.00–49.00)	57.00 (35.00–74.00)	< 0.001
GGT, U/L	24.00 (16.00–46.00)	23.50 (15.00–38.00)	0.545
ALB, g/L	40.20 (37.60–43.70)	40.10 (35.40–43.40)	0.393
TBIL, μmol/L	12.70 (8.90–16.70)	12.60 (9.50–19.00)	0.351
Lactate, mmol/L	1.80 (1.20–3.30)	3.05 (2.00–4.80)	< 0.001
INR	1.07 (0.99–1.17)	1.12 (1.03–1.24)	0.024
D-dimer, mg/L	2.78 (1.40–5.85)	18.24 (12.20–39.68)	< 0.001
DPR	14.24 (5.74–28.72)	108.54 (77.80–241.36)	< 0.001
Neurological evaluation			
GCS	6.00 (4.00–9.00)	4.00 (3.00–7.00)	< 0.001
Pupillary size, n (%)			> 0.999
Symmetry	90 (63.83)	62 (63.27)	
Asymmetry	51 (36.17)	36 (36.73)	
Complication			
Hypertension, n (%)			0.064
No	111 (78.72)	87 (88.78)	
Yes	30 (21.28)	11 (11.22)	
Diabetes, n (%)			0.975
No	128 (90.78)	88 (89.80)	
Yes	13 (9.22)	10 (10.20)	
Time of injury, hour	10.00 (4.00–19.00)	7.00 (3.00–11.00)	0.009
Outcomes			
Survival time, day	28.00 (28.00–28.00)	28.00 (5.00–28.00)	< 0.001
28-day Mortality, n (%)			< 0.001
Non-death	124 (87.94)	58 (59.18)	
Death	17 (12.06)	40 (40.82)	
END, n (%)	19 (13.77)	39 (41.05)	< 0.001

HR, Heart rate; RR, respiratory rate; MAP, mean arterial pressure; WBC, White Blood Cell; NEUT, neutrophil; LYM, lymphocyte; Hb, hemoglobin; PLT, platelet; ABG, admission blood glucose; ALT, alanine aminotransferase; AST, aspartate aminotransferase; GGT, gamma-glutamyl transferase; ALB, albumin; TBIL, total bilirubin; INR, international normalized ratio; DPR, D-dimer to Platelet Ratio; GCS, Glasgow Coma Scale; END, early neurological deterioration.

As shown in Table 4, the high DPR group presented with lower GCS scores (4 vs. 6, *P* = 0.001) and lower body temperature (36.7°C vs. 36.8°C, *P* = 0.005). In terms of laboratory evaluation, patients in the high group exhibited higher levels of ABG, ALT, AST, lactate, and INR (all *P* < 0.05), alongside lower platelet counts (160 vs. 201 10^9^/L, *P* < 0.001). Additionally, the high DPR group had a higher median age (56 vs. 52 years, *P* = 0.047) and a shorter time from injury to admission (7 vs. 10 h, *P* = 0.009). Finally, the incidence of 28-day mortality (40.82% vs. 12.06%, *P* < 0.001) and END (41.05% vs. 13.77%, *P* < 0.001) was significantly higher in the high DPR group.

### 3.5 Development and internal validation of the DPR-B risk stratification tool

**FIGURE 5:**
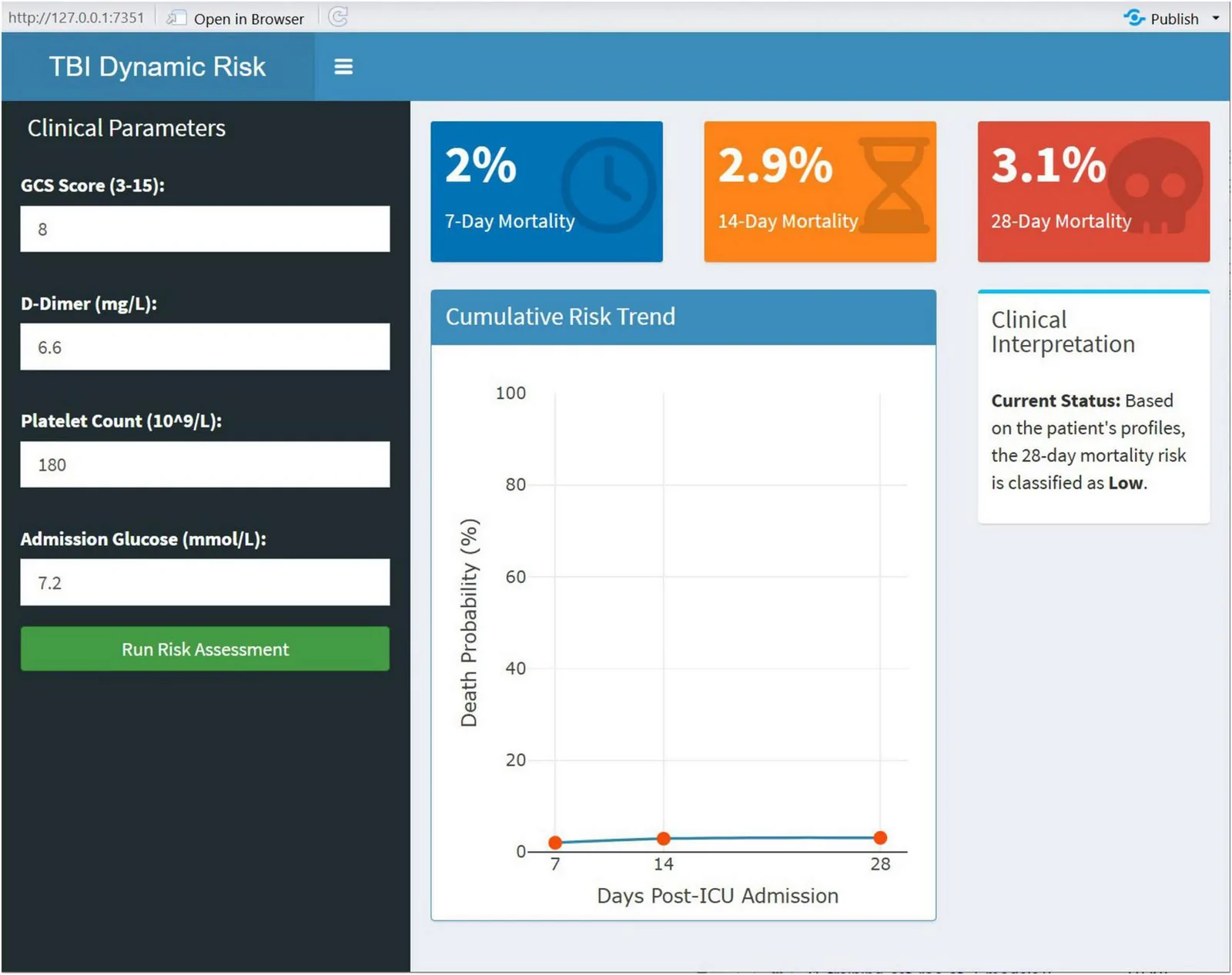
Interface of the online dynamic nomogram for illustrating the estimated 28-day mortality risk in patients with msTBI.

**FIGURE 6:**
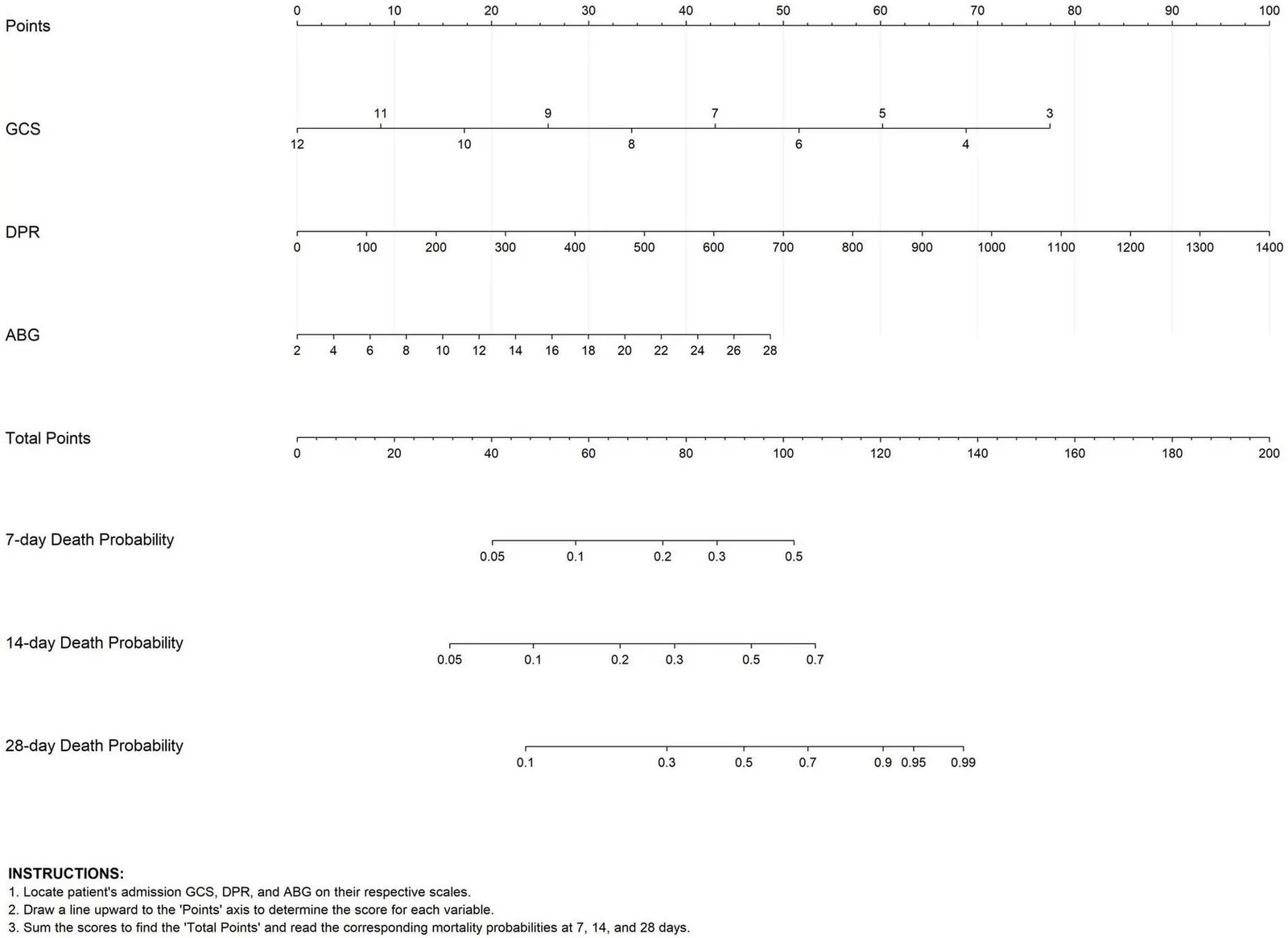
Static nomogram of the DPR-B model illustrating the estimated probability of 28-day all-cause mortality. Points for each variable are summed to obtain the total points, which correspond to the estimated probability of death at 7, 14, and 28 days.


Based on the identified prognostic factors, a risk stratification tool (DPR-B model) was developed. An online dynamic nomogram^1^ (Figure 5) and a static version (Figure 6) were constructed. The model is based on the following linear predictor (*LP*):

L⁢P=-0.361×GCS+0.003×DPR+0.079×ABG

The probability of mortality at a specific time *t* is calculated as *P*(*t*) = 1−*S*_0_(*t*)^*exp*(*LP*)^, where *S*_0_(*t*) is the baseline survival probability at time *t*. The coefficients are based on raw DPR values for practical nomogram calculation, whereas the HR reported in Table 3 corresponds to DPR standardized per 1 standard deviation increase.

As shown in the static nomogram (Figure 6), the model provides visual scales for estimating death probabilities at 7, 14, and 28 days. The model yielded a C-index of 0.815 and a 28-day time-dependent AUC of 0.852 (95% CI: 0.790–0.915).

**FIGURE 7:**
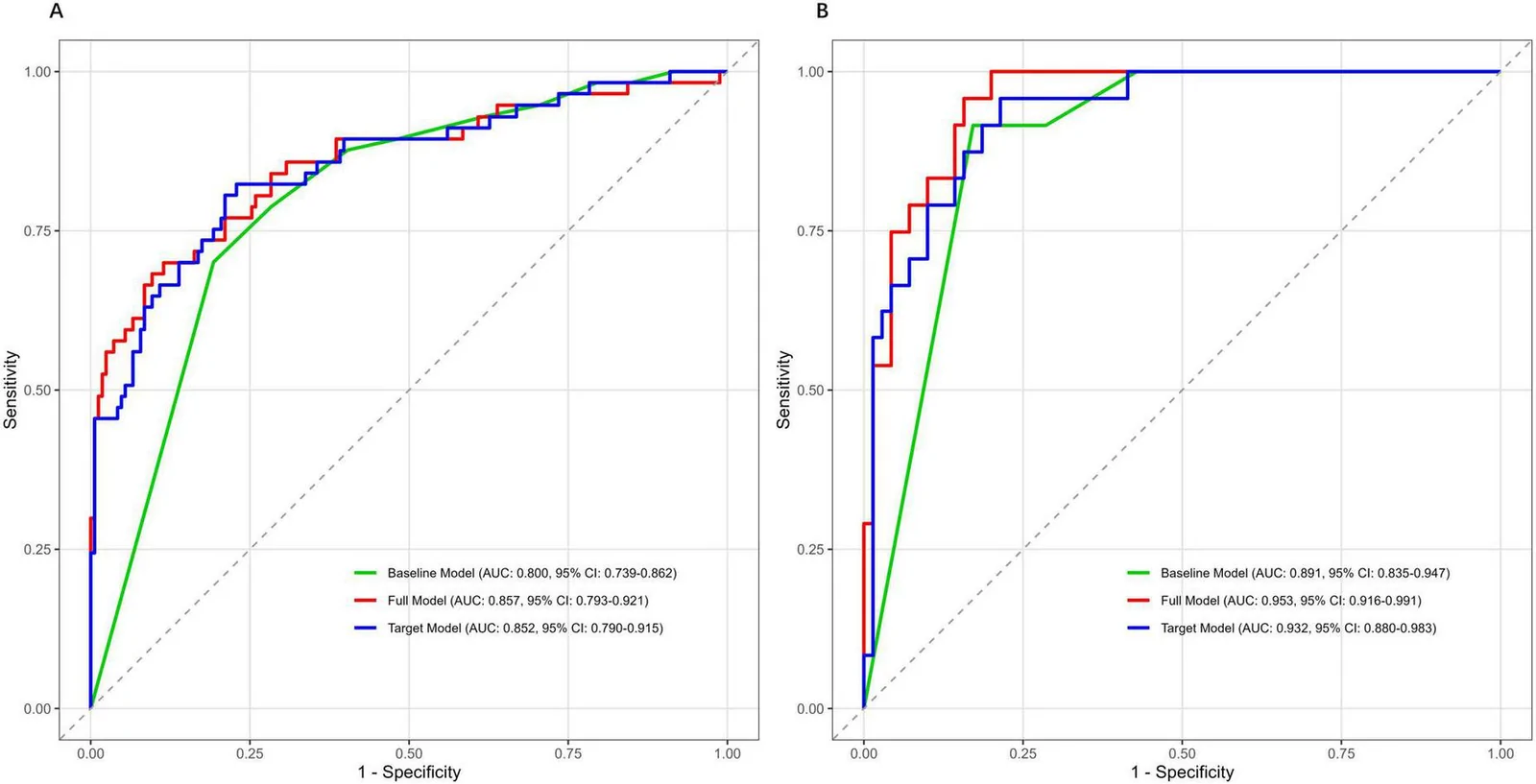
Comparison of time-dependent ROC curves for predicting 28-day mortality in msTBI patients. **(A)** The training cohort, and **(B)** the validation cohort. In each panel, the three models are distinguished by color: the DPR-B model (Target Model: GCS, DPR, and ABG) is shown in blue; the baseline model (GCS only) is shown in green; and the full model (GCS, DPR, ABG, Temperature, MAP, Sodium, and INR) is shown in red. ROC, receiver operating characteristic; msTBI, moderate-to-severe traumatic brain injury; GCS, Glasgow Coma Scale; ABG, admission blood glucose; AUC, area under the curve; CI, confidence interval.

The internal performance of the DPR-B model was evaluated using time-dependent ROC curves (Figure 7) and performance metrics (Table 5). In the training group, the 28-day AUC of the DPR-B model was 0.852, compared with 0.800 for the baseline GCS model (*P* = 0.001) and 0.857 for the full model (*P* = 0.582). Consistent discriminative ability was further confirmed in the split-sample validation group (AUC: 0.932, 95% CI: 0.880–0.983). The AIC and Brier scores for the DPR-B model were 530.26 and 0.1142, respectively.

**TABLE 5:** Predictive performance metrics of different models in the training and validation groups.

Group	Model	C-index	28-day AUC	AIC	Brier score
Training group (*n* = 239)	Baseline model	0.759	0.800	560.29	0.1423
	DPR-B model	0.815	0.852	530.26	0.1142
	Full Model	0.819	0.857	533.20	0.1083
Validation group (*n* = 101)	Baseline model	0.849	0.852	–	0.1219
	DPR-B model	0.890	0.857	–	0.0984
	Full Model	0.915	0.891	–	0.1004

Baseline model: GCS only. DPR-B model: GCS + DPR + ABG. Full model: GCS + DPR + ABG + Temperature + MAP + Sodium + INR. AUC, area under the curve; AIC, Akaike information criterion; GCS, Glasgow Coma Scale; DPR, D-dimer to platelet ratio; ABG, admission blood glucose; MAP, mean arterial pressure; INR, international normalized ratio.

The reclassification improvement of the DPR-B model was quantified using NRI and IDI (Table 6). Compared with the baseline model, the DPR-B model showed reclassification improvement in both the training group (NRI: 0.470, *P* = 0.008; IDI: 0.101, *P* < 0.001) and the validation group (NRI: 0.530, *P* = 0.012; IDI: 0.134, *P* < 0.001). When comparing the full model to the DPR-B model in the training group, the NRI was 0.057 (*P* = 0.680) and the IDI was 0.015 (*P* = 0.256).

**TABLE 6:** Statistical comparison of discriminative ability and reclassification improvement.

Comparison	Training group			Validation group		
	AUCs (P)	NRI (P)	IDI (P)	AUCs (P)	NRI (P)	IDI (P)
DPR-B vs. Baseline model	0.852 vs. 0.800 (0.001)	0.470 (0.008)	0.101 ( < 0.001)	0.932 vs. 0.891 (0.157)	0.530 (0.012)	0.097 (0.012)
DPR-B vs. Full model	0.852 vs. 0.857 (0.582)	0.057 (0.680)	0.015 (0.256)	0.932 vs. 0.953 (0.199)	0.065 (0.316)	0.031 (0.216)
Baseline vs. Full model	0.800 vs. 0.857 (0.001)	0.334 (0.020)	0.116 ( < 0.001)	0.891 vs. 0.953 (0.027)	0.150 (0.328)	0.128 ( < 0.001)

PR-B model: GCS + DPR + ABG. Baseline model: GCS only. Full model: GCS + DPR + ABG + Temperature + MAP + Sodium + INR. AUC, area under the curve; NRI, net reclassification improvement; IDI, integrated discrimination improvement; GCS, Glasgow Coma Scale; DPR, D-dimer to platelet ratio; ABG, admission blood glucose.

**FIGURE 8:**
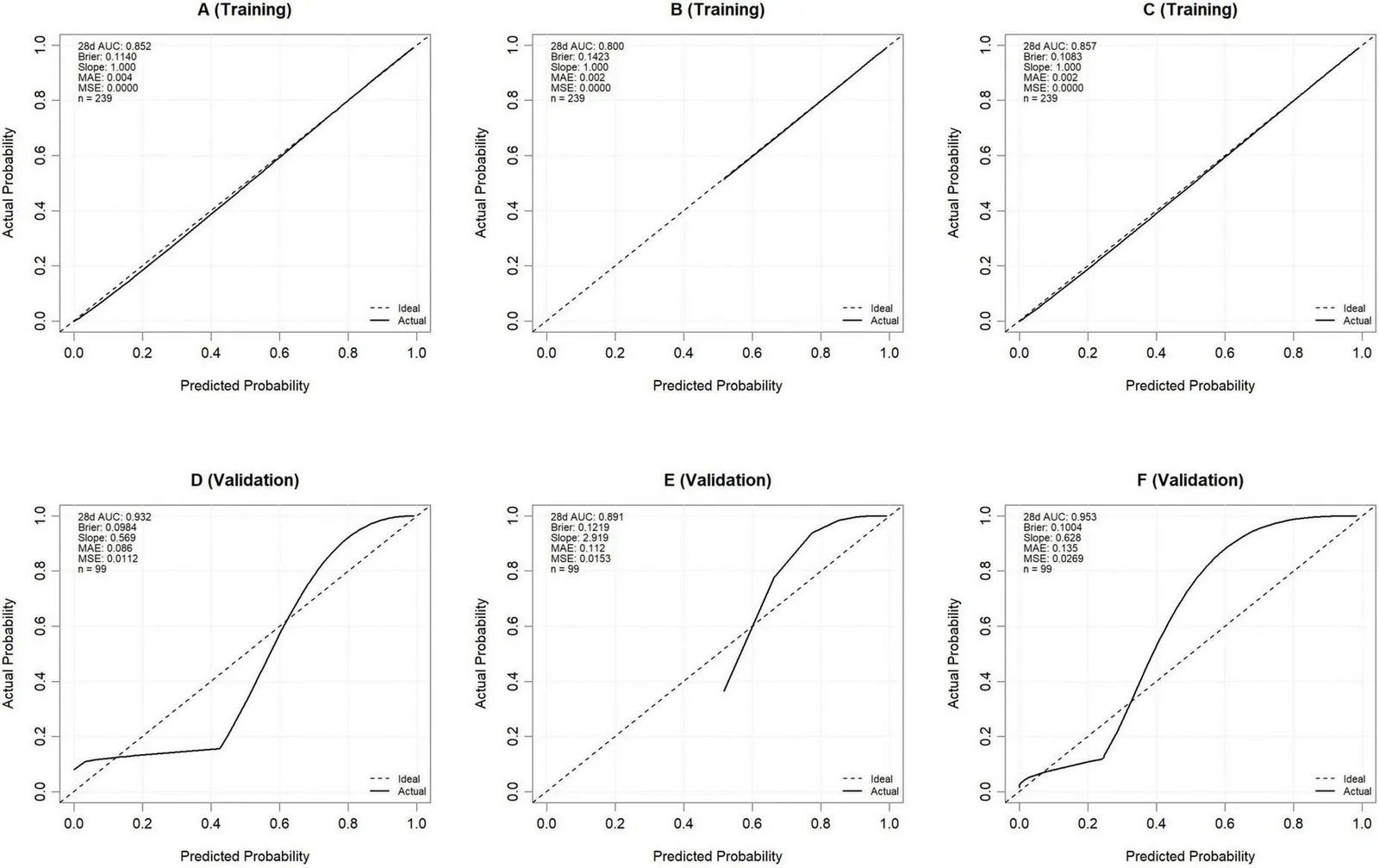
Calibration curves of different models for predicting 28-day all-cause mortality. **(A–C)** The training group, and **(D–F)** the validation group. **(A,D)** The DPR-B model; **(B,E)** baseline model (GCS only); **(C,F)** full model. The dashed line represents the ideal prediction, and the solid line represents the actual performance of each model. The Brier score, Mean Absolute Error (MAE), and Mean Squared Error (MSE) for each model are provided within the respective panels.

Calibration curves were utilized to assess the agreement between the predicted and observed 28-day all-cause mortality (Figure 8). In the training cohort (Figures 8A–C), the predicted probabilities for the Target, Baseline, and Full models closely aligned with the observed outcomes. In the validation cohort (Figures 8D–F), all models deviated from the ideal diagonal reference line, indicating differences between predicted risk and actual mortality across the range of probability.

To evaluate the robustness of the DPR-B model, we performed a sensitivity analysis comparing the raw DPR formulation against alternative specifications, including log_10_- DPR, categorized DPR, log_10_- D-dimer, D-dimer, platelet count, and their combinations (Supplementary Table S7 and Supplementary Figures S6–S8). The raw DPR model yielded an AUC of 0.852 and an AIC of 530.26, exhibiting higher AUC and lower AIC values than all other alternative specifications tested. Furthermore, reclassification analyses (NRI and IDI) indicated that none of the alternative formulations consistently provided statistically significant improvements in predictive ability over the raw DPR model in both training and validation cohorts (Supplementary Table S8). When compared with the Full Model (AUC: 0.857, AIC: 533.20), the raw DPR model utilized fewer variables (3 vs. 7) while maintaining similar discriminative performance. These data suggest that the raw DPR formulation provides a balance between predictive accuracy and model complexity.

**FIGURE 9:**
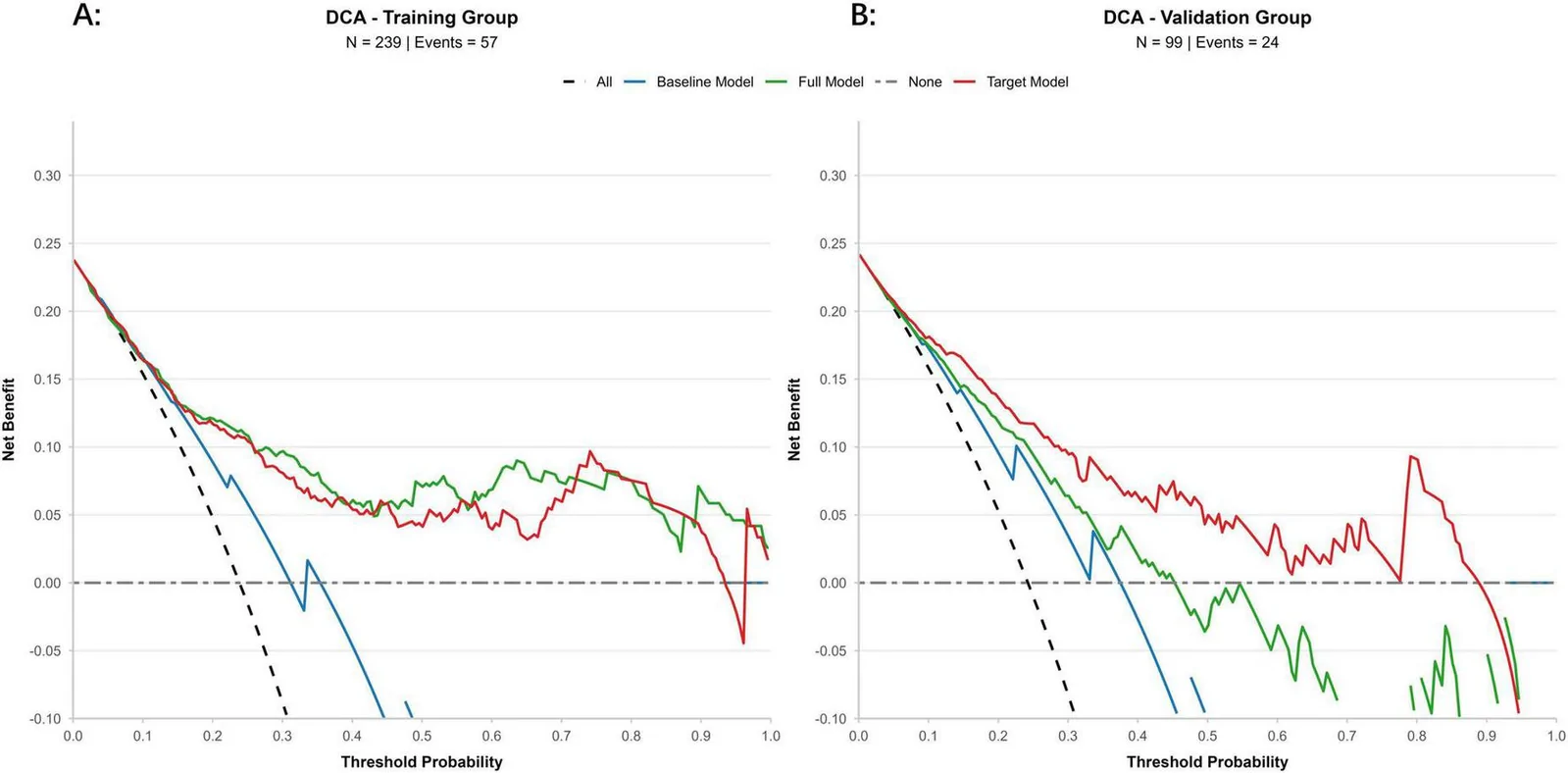
Decision curve analysis (DCA) for predicting 28-day mortality in patients with msTBI. The y-axis represents the net benefit, and the x-axis represents the threshold probability. **(A)** Training group (*N* = 239); **(B)** validation group (*N* = 101). DPR-B model: GCS + DPR + ABG; Baseline model: GCS only; Full model: GCS + logDPR + ABG + Temperature + MAP + Sodium + INR.

Decision curve analysis (DCA) evaluated the clinical utility of the models (Figure 9). Within the clinically relevant threshold range of 0.10–0.50, the Target Model (DPR-B) consistently showed higher net benefit than the Baseline Model and the default strategies of treating all or none in both cohorts. In the split-sample validation cohort, the Target Model also demonstrated higher net benefit than the Full Model across a broader threshold range (0.10–0.85). At a threshold probability of 0.20, the Target Model achieved a net benefit of 0.142 in the split-sample validation cohort, compared with 0.125 for the Full Model and 0.098 for the Baseline Model. These findings suggest that the Target Model offers a favorable balance between parsimony and clinical utility, with less evidence of overfitting than the Full Model. The Target Model yielded positive net benefit across the threshold probability range in both the training and split-sample validation cohorts and exceeded the Baseline model. In the split-sample validation cohort at a threshold probability of 0.2, the Target Model showed higher net benefit than the Full Model (Difference: 0.0204; 95% CI: 0.0076–0.0354; *P* < 0.001). The Full Model included more variables but showed lower net benefit in the validation cohort, which suggests potential overfitting. The Target Model maintained higher net benefit with fewer variables.

### 3.6 Association of DPR with early neurological deterioration (END)

Having established that DPR is independently associated with 28-day mortality, we next examined whether it is also associated with a more proximal endpoint—early neurological deterioration (END)—to validate its mechanistic relevance to post-traumatic coagulopathy.

Among the 239 patients in the training group, 6 patients (2.5%) were excluded from the END analysis because they had no follow-up CT and no clinical deterioration (no GCS decline, no pupillary abnormality, and did not die within 72 h). The remaining 233 patients were included in the analysis. In the validation group, 2 patients (2.0%) were excluded for the same reason, leaving 99 patients for analysis. In the training group, the overall rate of missing follow-up CT within 72 h was 10.04% (Supplementary Table S9), with higher missing rates among patients who died within 72 h (53.85%) compared with those with late death ( > 72h) (25.81%, *P* = 0.030). Detailed missing CT rates for the total cohort, training set, and validation set are provided in Supplementary Table S9.

Multivariable logistic regression was performed to identify independent predictors of END. Candidate variables were selected using the Boruta algorithm, consistent with the primary analysis. After adjusting for potential confounders (temperature, MAP, GCS, ABG, INR, DPR, and age), DPR remained significantly associated with END (OR: 2.223, 95% CI: 1.367–3.614, *P* = 0.001), along with GCS (OR: 0.853, 95% CI: 0.740–0.984, *P* = 0.029) and ABG (OR: 1.127, 95% CI: 1.022–1.243, *P* = 0.017) (Supplementary Table S10). The area under the receiver operating characteristic curve (AUC) for DPR alone in predicting END was 0.724 (95% CI: 0.643–0.806) (Supplementary Figure S9). The discriminative ability of DPR for END was further assessed in the validation cohort, yielding an AUC of 0.874 (95% CI: 0.786–0.962) (Supplementary Figure S10). To assess whether the predictive association between DPR and END was influenced by the inclusion of early mortality, a sensitivity analysis was performed in the training cohort, excluding patients who died within 72 h (*n* = 206). In this subset, DPR remained significantly associated with END (OR: 1.869, 95% CI: 1.049–3.329, *P* = 0.0337; Supplementary Table S11). These findings indicate that the association between DPR and END is not solely attributable to early mortality.

Of note, the independent predictors of END (GCS, ABG, and DPR) were the same as those for 28-day mortality in the primary Cox model. This finding suggests that DPR may capture a common pathophysiological pathway linking early neurological worsening and longer-term survival.

## 4 Discussion

This retrospective study developed and evaluated a parsimonious risk stratification tool, the DPR-B model, utilizing Cox proportional hazards regression to estimate the risk of 28-day all-cause mortality in 340 patients with msTBI. The model incorporates three readily available parameters: the GCS, ABG, and DPR. In the current analysis, the model demonstrated a 28-day AUC of 0.852 in the training group, with comparable discriminative ability and calibration observed in the split-sample validation group. While 28-day all-cause mortality is a metric for evaluating overall survival and ICU resource allocation, it represents a distal outcome influenced by various post-admission confounders. Notably, our exploratory analysis (section 3.6) confirmed that the same core variables—GCS, ABG, and DPR—were independently associated with END (Vásquez-García et al., 2025), a more proximal clinical event (Fletcher-Sandersjöö et al., 2023; Mohammadzadeh et al., 2025). This alignment indicates that the DPR-B score captures a pathological signal during the acute phase that contributes to subsequent clinical progression, suggesting a potential link between initial hemostatic status and 28-day survival.

Our findings indicate that the GCS score is independently associated with the 28-day mortality hazard in patients with msTBI (HR: 0.705, 95% CI: 0.596–0.835, *P* = 0.001). Previous research has demonstrated that the GCS remains a primary tool for neurological assessment, where lower scores reflect the severity of initial mechanical brain injury and correlate with mortality risk (Gorji et al., 2014; Maas et al., 2022). The utility of this metric stems from its design, which enables objective evaluation of a patient’s level of consciousness and neurological responsiveness (Teasdale and Jennett, 1974; Reith et al., 2016). Large-scale collaborations, including the TRACK-TBI project (Steyerberg et al., 2019) and the CENTER-TBI registry in both European and Chinese cohorts (McCrea et al., 2021; Lang et al., 2023), have utilized the GCS as a core variable for risk stratification across diverse patient populations. As highlighted in the consensus derived from these datasets, the GCS serves as a clinical surrogate for injury magnitude, reflecting the extent of underlying pathology such as diffuse axonal injury or brainstem compromise (Maas et al., 2022). In our model, the predictive value of the GCS aligns with these observations and indicates that the relationship between initial neurological status and the hazard of 28-day mortality remains stable even within a time-to-event analysis framework.

Admission Blood Glucose (ABG) was identified as an independent predictor of the 28-day mortality hazard in our model (HR: 1.068, 95% CI: 1.002–1.139, *P* = 0.044). This finding is consistent with the observation that admission hyperglycemia reflects the systemic stress response in trauma (Rau et al., 2017). Similarly, meta-analytic evidence has indicated that stress-induced hyperglycemia is associated with increased mortality in TBI patients, independent of injury severity (Cui et al., 2025). Previous studies have investigated the pathological mechanisms involved; for instance, hyperglycemia may act synergistically with pro-inflammatory cytokines to exacerbate neuroinflammation in msTBI (Al-Hassani et al., 2024). Beyond its role as a clinical marker, hyperglycemia contributes to pathophysiology. Under conditions of post-traumatic cerebral ischemia, glucose overload exacerbates metabolic dysregulation, triggering mitochondrial dysfunction—a known contributor to neurodegeneration (Olatona et al., 2025). As mitochondrial impairment is a driver of secondary energy failure that influences outcomes (Hubbard et al., 2021), the independent predictive capability of ABG in our current cohort likely reflects the impact of this metabolic disturbance on the mortality hazard over the 28-day clinical window.

The DPR was identified as an independent prognostic factor for the 28-day mortality hazard, reflecting the admission hemostatic status in patients with msTBI. The prognostic relevance of DPR stems from its association with proximal pathological changes, mechanistically linked to progressive hemorrhagic injury (PHI) and clinically manifested as END (Fletcher-Sandersjöö et al., 2024; Mohammadzadeh et al., 2025). In our exploratory analysis (section 3.6), elevated DPR was independently associated with an increased risk of END, suggesting that this ratio acts as a clinical indicator of early coagulation imbalance that directly precedes neurological worsening. The definition of END includes death within 72 h, which results in partial overlap with 28-day mortality. While this introduces potential multicollinearity, excluding patients who died early could introduce selection bias. To address this, a sensitivity analysis was performed excluding patients who died within 72 h. In this subset, the association between DPR and non-fatal END remained statistically significant (*P* = 0.0337). These findings suggest that the predictive value of DPR for early neurological deterioration is not dependent on the occurrence of early death. This prognostic value derives from its capacity to assess the imbalance between two synchronous pathological pathways: fibrinolysis activation and consumptive coagulopathy.

First, D-dimer serves as a surrogate for fibrinolytic activation secondary to the release of tissue factor (TF) and brain-derived extracellular vesicles (BDEVs) from the injured brain (Moore et al., 2021; Nakae et al., 2022a). Upon blood-brain barrier disruption, TF initiates the extrinsic coagulation pathway, followed by a rise in fibrinolysis that typically peaks within 3 h post-injury (Wada et al., 2017; Moore et al., 2021; Nakae et al., 2022b). Circulating BDEVs reach peak levels at approximately the same interval, carrying procoagulant TF that triggers a systemic hypercoagulable state followed by secondary hyperfibrinolysis (Wang et al., 2022; Li et al., 2024). In our cohort, elevated D-dimer levels likely reflect this BDEV-mediated fibrinolytic activity, which is a recognized risk factor for PHI (Yuan et al., 2016; Chen et al., 2023; Mohammadzadeh et al., 2025). Previous studies have indicated that admission D-dimer elevation correlates with complications and poor neurological outcomes (Xu et al., 2020; Fujiwara et al., 2022; Chen et al., 2023). Furthermore, D-dimer levels have been utilized to assess the risk of structural disorders and the potential requirement for early airway management (Sugimoto et al., 2017; Minoza et al., 2025). Meta-analytic evidence indicates that early hyperfibrinolysis facilitates contusion expansion, which influences survival over the clinical course (Fletcher-Sandersjöö et al., 2023, 2024).

Second, the platelet count reflects the consumptive aspect of TIC. Our results showed lower platelet counts in the mortality group, aligning with findings that thrombocytopenia correlates with preoperative hematoma expansion, particularly in acute subdural hematoma (Thakkar et al., 2022; Yuguero et al., 2023). Literature suggests that thrombocytopenia is associated with mortality, especially in elderly populations or those with severe injury (Yuguero et al., 2023; Zhang et al., 2023). The reduction in platelets is multifactorial, involving consumption at the injury site, hemodilution, and dysfunction following initial activation (Fletcher-Sandersjöö et al., 2021; Hubbard et al., 2021; Rachel et al., 2025). This process is increasingly linked to BDEV-platelet interactions, where BDEVs activate platelets and facilitate their clearance from circulation (Li et al., 2024). This mechanism contributes to both the decline in platelet count and potential systemic inflammatory responses involving extra-cranial organs (Gao et al., 2022; Li et al., 2023).

This BDEV-mediated cascade provides a pathophysiological framework for how an admission measurement correlates with distal outcomes: it captures the initiation of a systemic response extending from the initial neurological injury. Consequently, the DPR serves as a functional index of hemostatic decompensation. In our study, the DPR cut-off of 54.339 identifies a high-risk phenotype characterized by excessive fibrinolysis and platelet consumption. For context, a patient with a D-dimer of 5.5 mg/L and a platelet count of 100 × 10^9^/L would exceed this threshold, suggesting a higher risk for clinical progression. By assessing the imbalance between clot degradation and remaining hemostatic capacity, the DPR provides a more sensitive indicator of risk than single biomarkers. This aligns with the reported prognostic value of DPR in other critical conditions, including sepsis (Zhao et al., 2023), acute aortic dissection (Zhao et al., 2024), and decompensated cirrhosis (He and Ding, 2024). Collectively, these findings support the use of DPR for risk stratification in msTBI.

The exploratory DPR-B model demonstrated a 28-day AUC of 0.852 in the training group, showing discriminative performance comparable to recent large-scale TBI mortality studies, such as the CENTER-TBI China registry-based nomogram (AUC 0.856) (Lang et al., 2023) and a 2025 multicenter prognostic tool (training set 28-day AUC 0.832) (Wu and Li, 2025). Although the initial model constructed from our complete set of candidate variables resulted in a slightly higher AUC (0.857), the difference was not statistically significant (*P* = 0.582). This choice aligns with the principle of parsimony, favoring a simplified three-variable tool to balance predictive performance with clinical feasibility in acute trauma settings. Indeed, systematic reviews of machine learning-based models in TBI have reported discriminative power with AUC values ranging from 0.72 to 0.96 and a pooled AUC of 0.90 (Wu et al., 2023); however, our results suggest that an approach utilizing GCS, ABG, and DPR can achieve competitive accuracy without the need for complex computational algorithms. It should be noted that while TBI prognostic models frequently achieve high discrimination, their performance and calibration can be inconsistent across different cohorts (Eagle et al., 2023). The incremental value of adding DPR and ABG to the GCS was supported by a Net Reclassification Improvement (NRI) of 0.470 and an Integrated Discrimination Improvement (IDI) of 0.101 (*P* < 0.05), indicating a refinement in risk reclassification. While universal scoring systems like APACHE II are utilized, evidence suggests they may not offer improved predictive accuracy over neuro-specific tools in head injury populations (Hosseini et al., 2016; Nik et al., 2018). Notably, unlike those externally validated models, the DPR-B model has been evaluated only through internal split-sample validation. The observed performance in the validation group may therefore be optimistic and requires confirmation in external, independent cohorts

The potential clinical relevance of the DPR-B model was explored via Decision Curve Analysis (DCA). By defining the decision scenario as risk identification for patients at risk of clinical progression, the DCA demonstrated a net benefit across a range of threshold probabilities (Weng et al., 2019; Lawati et al., 2021). Within the clinically relevant threshold range of 0.10–0.50, the Target Model consistently showed higher net benefit than the Baseline Model and the default strategies in both cohorts. At a threshold probability of 0.20, for instance, the Target Model achieved a net benefit of 0.142 in the validation cohort, compared with 0.125 for the Full Model and 0.098 for the Baseline Model. These findings suggest that the Target Model offers a favorable balance between parsimony and clinical utility, with less evidence of overfitting than the Full Model.

The DCA results can be translated into potential clinical workflows. Taking a pragmatic threshold of 0.20 as an example, patients identified as high-risk could be prioritized for intensified hemostatic monitoring, including serial coagulation assessments, and timely repeat neuroimaging such as urgent follow-up head CT to detect early structural deterioration, as well as prioritized triage to a neuro-intensive care unit or high-dependency unit. For patients below this threshold, conservative observation may be sufficient, which could help reduce unnecessary CT exposure and optimize ICU bed utilization. Thus, the model provides a quantitative approach for risk stratification, connecting admission status with 28-day survival. While recent research has developed pre-hospital prediction models for TBI mortality (Wang et al., 2024), the DPR-B tool serves as a complementary instrument for identifying patients who may require hemostatic monitoring upon ICU admission. For instance, the DPR level at admission could serve as a signal for identifying patients at risk of hemostatic decompensation, particularly in cases where clinical worsening is anticipated. Finally, the online tool provides risk estimates that may support studies on ICU resource allocation.

This study has several limitations. First, 28-day all-cause mortality is a distal endpoint influenced by various post-admission confounders, such as surgical interventions and infectious complications, which may weaken its direct link to admission coagulopathy. To address this, we conducted an exploratory analysis using early neurological deterioration (END) as a more proximal outcome. The finding that the variables in the exploratory DPR-B model—GCS, ABG, and DPR—remained independently associated with END (section 3.6) provides preliminary evidence of the mechanistic relevance of admission hemostatic status to clinical progression. Second, the retrospective design of this study may involve selection bias and precludes the determination of causation. While multiple factors were accounted for, unmeasured variables—including specific neuroimaging findings and injury subtypes—could influence the results. Third, this study was conducted at a single center, which may limit the generalizability of the proposed tool to other clinical settings. The split-sample validation within a single center does not constitute external validation and limits generalizability. The observed baseline imbalance in certain variables, such as INR (*P* = 0.022), further underscores the necessity for future multicenter prospective validation. Notably, TBI prognostic models are known to exhibit variability in performance and calibration when applied to different populations or healthcare settings (Eagle et al., 2023; Behzadi et al., 2024). For instance, machine learning-based models have shown significant differences in predictive performance between high-income and low- and middle-income countries (Warman et al., 2022). Therefore, external validation of the DPR-B model in diverse cohorts—including different geographic regions and healthcare systems—is essential before any clinical application can be considered. Fourth, DPR was measured only at admission, which does not capture the dynamic evolution of post-traumatic coagulopathy. Incorporating serial DPR measurements in future research could provide more nuanced prognostic insights. Fifth, post-admission interventions, such as surgical procedures, blood transfusions or the non-standardized use of tranexamic acid (TXA), were not controlled and may act as effect modifiers. It is possible that elevated DPR levels influenced clinical decisions regarding these interventions, which in turn could have affected mortality outcomes. As such, the observed association between admission DPR and 28-day mortality may partly reflect downstream treatment effects rather than a direct prognostic effect alone. Finally, while the DPR-B model prioritized parsimony for practical application, the higher numerical AUC of the full model suggests that excluding certain parameters might lead to oversimplification.

## 5 Conclusion

In summary, admission DPR is independently associated with both early neurological deterioration and 28-day mortality in patients with msTBI. Adding ABG and DPR to the GCS may assist in early risk stratification. These findings are exploratory and require external validation in prospective multi-center studies before any clinical translation can be considered.

## Data Availability

The raw data supporting the conclusions of this article will be made available by the authors, without undue reservation.

## Footnotes

1
https://zhaoyiwang20251123.shinyapps.io/DPR_TBI/
