# Supplementary material for: Development and validation of a risk stratification tool for 28-day mortality based on D-dimer to platelet ratio in moderate-to -severe traumatic brain injury: a retrospective cohort study
Source: Front Neurosci. 2026 Aug 11;20:1908494. doi: 10.3389/fnins.2026.1908494 (PMC13503328; doi:10.3389/fnins.2026.1908494)
Supplement: Supplementary file 1 [file Data_Sheet_1.docx]

**Supplementary Fig. 1. Distribution of D-dimer to Platelet Ratio (DPR) before and after logarithmic transformation**

**Note: Panel A:** The original distribution of DPR in the study cohort. The data exhibits significant right skewness (Skewness = 3.803), with a mean of 92.81 and a median of 40.68. **Panel B:** The distribution of DPR after log10 transformation. The transformation successfully addresses the right skewness and improves the normality of the data (Mean = 1.537, Median = 1.609), making it suitable for subsequent survival analysis. The orange and blue dashed lines indicate the mean and median of the distributions, respectively.

**Supplementary Fig. 2. Distribution of D-dimer before and after logarithmic transformation**

**Note: Panel A:** The original distribution of D-dimer levels at admission. Similar to DPR, the raw D-dimer values are right-skewed (Skewness = 2.145), with a mean of 13.06 mg/L and a median of 6.57 mg/L. **Panel B:** The distribution of log10-transformed D-dimer. The transformation results in a more symmetric distribution (Mean = 0.774, Median = 0.818), satisfying the assumptions of the Cox proportional hazards model. **Note:** The orange and blue dashed lines represent the mean and median, respectively. The D-dimer unit used in the original distribution is mg/L.

**Supplementary Fig. 3. Spearman Correlation Matrix of Candidate Prognostic Factors**

**Supplementary Fig. 3.** Spearman correlation heatmap displaying the relationships between candidate prognostic factors for 28-day mortality. Correlation coefficients are presented within each cell, with blue indicating positive correlations, orange indicating negative correlations, and color intensity reflecting the strength of the correlation. The correlation coefficient between D-dimer and log10-DPR is 0.77, indicating interdependency between these two variables.

**Supplementary Table 1. Summary of Missing Data for Variables in the Final Analytical Cohort (n = 340)**

| **Variable Name** | **Total Assessed (n)** | **Missing (n)** | **Missing Rate (%)** |
| --- | --- | --- | --- |
| Potassium | 340 | 5 | 1.47% |
| ALB | 340 | 5 | 1.47% |
| Calcium | 340 | 4 | 1.18% |
| ABG | 340 | 1 | 0.29% |
| Sodium | 340 | 1 | 0.29% |
| Chloride | 340 | 1 | 0.29% |
| Temperature | 340 | 0 | 0.00% |
| HR | 340 | 0 | 0.00% |
| RR | 340 | 0 | 0.00% |
| MAP | 340 | 0 | 0.00% |
| GCS | 340 | 0 | 0.00% |
| day_28_ACM | 340 | 0 | 0.00% |
| survival_time | 340 | 0 | 0.00% |
| age | 340 | 0 | 0.00% |
| gender | 340 | 0 | 0.00% |
| Time_of_injury | 340 | 0 | 0.00% |
| History_of_hypertension | 340 | 0 | 0.00% |
| Previous_diabetes_treatment | 340 | 0 | 0.00% |
| Pupillary_size | 340 | 0 | 0.00% |
| WBC | 340 | 0 | 0.00% |
| Hb | 340 | 0 | 0.00% |
| PLT | 340 | 0 | 0.00% |
| Urea | 340 | 0 | 0.00% |
| Creatinine | 340 | 0 | 0.00% |
| ALT | 340 | 0 | 0.00% |
| AST | 340 | 0 | 0.00% |
| GGT | 340 | 0 | 0.00% |
| TBIL | 340 | 0 | 0.00% |
| Lactate | 340 | 0 | 0.00% |
| INR | 340 | 0 | 0.00% |
| D_Dimer | 340 | 0 | 0.00% |
| DPR | 340 | 0 | 0.00% |
| NEUT | 340 | 0 | 0.00% |
| LYM | 340 | 0 | 0.00% |

**Note:** Missing values include NAs, empty strings, and 'Unknown' entries in the records. Core predictors used in the final multivariable Cox model (GCS, ABG, and log10-DPR) exhibited a high degree of completeness, with missingness ≤ 0.3%.

**Supplementary Fig. 4. Dose-response relationship between clinical variables and 28-day mortality risk.**

**Supplementary Fig. 4. Dose-response relationship between clinical variables and 28-day mortality risk.** Restricted cubic spline (RCS) models were used to visualize the association between potential risk factors and 28-day all-cause mortality: (A) DPR, (B) D-Dimer, (C) log_10_-D-Dimer, and (D) PLT. Solid blue lines represent the hazard ratios (HR), and shaded areas represent the 95% confidence intervals (CI). The horizontal red dashed line indicates a HR of 1.0. The corresponding *P*-total and *P*-nonlinear values are presented at the top of each panel. Abbreviations: DPR, D-dimer to platelet ratio; PLT, platelet; HR, hazard ratio; CI, confidence interval.

**Supplementary Fig. 5. Non-linear association between log10-transformed DPR and 28-day mortality.**

**Supplementary Fig. 5. Non-linear association between log10-transformed DPR and 28-day mortality.** Restricted cubic spline (RCS) model demonstrating the non-linear relationship between log_10_-DPR and the hazard ratio (HR) for 28-day all-cause mortality (*P*-nonlinear = 0.0076). The blue solid line represents the HR, and the shaded area indicates the 95% CI. The red dashed line denotes a reference HR of 1.0. Abbreviations: DPR, D-dimer to platelet ratio.

**Supplementary Table 2. Performance comparison of different functional forms of DPR in predicting 28-day all-cause mortality.**

| **Model Specification** | **AIC** | **C-index** | **Global PH *P*-value** |
| --- | --- | --- | --- |
| Raw DPR | 533.20 | 0.8187 | 0.3128 |
| Categorized DPR | 547.88 | 0.8095 | 0.7526 |
| log_10_-DPR (RCS) | 537.6 | 0.8183 | 0.4269 |
| D-dimer | 540.44 | 0.8155 | 0.5858 |
| log_10_-D-Dimer | 544.07 | 0.8103 | 0.484 |
| PLT | 545.44 | 0.8067 | 0.5587 |
| D-dimer + PLT | 539.19 | 0.8111 | 0.6292 |

**Note:** These analyses were performed to identify the optimal functional form of DPR for the final multivariable Cox regression model. **Abbreviations:** AIC, Akaike information criterion; PH, proportional hazards; DPR, D-dimer/platelet ratio; PLT, platelet count; RCS, restricted cubic splines.

**Supplementary Table 3. Sensitivity analysis of the prognostic value of DPR using winsorization.**

| **Analysis Approach** | **Hazard Ratio (HR)** | **Relative Change (%)** |
| --- | --- | --- |
| Original model (Scaled DPR) | 1.522 | - |
| Winsorized model (95th percentile) | 1.449 | 4.80% |

**Footnote:** Abbreviations: HR, hazard ratio; DPR, D-dimer/platelet ratio. The original model included scaled DPR as a continuous variable. The winsorized model utilized the 95th percentile to limit extreme values. Relative change was calculated as: |HR_winsorized − HR_original| / HR_original × 100.

**Supplementary Table 4. Proportional hazards assumption testing based on Schoenfeld residuals for the multivariable Cox model**

| **Variable** | **Chisq** | **df** | **P-value** |
| --- | --- | --- | --- |
| Temperature | 2.5186 | 1 | 0.11 |
| Mean Arterial Pressure (MAP) | 0.5349 | 1 | 0.46 |
| Glasgow Coma Scale (GCS) | 0.0337 | 1 | 0.85 |
| Admission Blood Glucose (ABG) | 1.2569 | 1 | 0.26 |
| Sodium | 2.2277 | 1 | 0.14 |
| INR | 0.4156 | 1 | 0.52 |
| DPR | 2.0775 | 1 | 0.15 |
| **GLOBAL** | **8.2294** | **7** | **0.31** |

**Note:** A P-value > 0.05 indicates that the proportional hazards assumption is satisfied for the multivariable Cox model.

**Supplementary Table 5. Univariable and Multivariable Logistic Regression Analysis of 28-Day All-Cause Mortality as a Sensitivity Analysis**

| **Variable** | **Univariable Analysis** |  | **Multivariable Analysis** |  |
| --- | --- | --- | --- | --- |
|  | **OR (95% CI)** | **P-value** | **OR (95% CI)** | **P-value** |
| **Temperature** | 0.514 (0.306–0.816) | 0.004 | 0.677 (0.391–1.119) | 0.143 |
| **MAP** | 0.984 (0.970–0.999) | 0.035 | 0.997 (0.980–1.014) | 0.752 |
| **GCS** | 0.600 (0.487–0.714) | <0.001 | 0.678 (0.549–0.809) | <0.001 |
| **ABG** | 1.240 (1.139–1.360) | <0.001 | 1.154 (1.038–1.295) | 0.011 |
| **INR** | 20.705 (4.200–122.603) | <0.001 | 5.908 (1.016–56.332) | 0.081 |
| **DPR (per 1 SD)** | 3.513 (2.301–5.768) | <0.001 | 2.201 (1.413–3.691) | 0.001 |
| **Age** | 1.015 (0.995–1.035) | 0.146 | — | — |
| **Sodium** | 1.059 (0.998–1.126) | 0.059 | — | — |
| **Creatinine** | 1.004 (0.999–1.011) | 0.142 | — | — |
| **D-Dimer (per 1 SD)** | 2.509 (1.835–3.543) | <0.001 | — | — |

**Note**: Logistic regression was performed as a sensitivity analysis. Multivariable analysis included variables with *P* < 0.05 in univariable Cox regression or those with established clinical relevance. This confirms the robust prognostic value of the DPR-B model components (GCS, ABG, and DPR). **Abbreviations**: OR, odds ratio; CI, confidence interval; MAP, mean arterial pressure; GCS, Glasgow Coma Scale; ABG, admission blood glucose; INR, international normalized ratio; DPR, D-dimer to platelet ratio.

**Supplementary Table 6. Predictive performance of the optimal log_10_-DPR cut-off in the training and validation cohorts**

| **Metric** | **Training Cohort (N=239)** | **Validation Cohort (N=101)** |
| --- | --- | --- |
| **Optimal Cut-off** | 54.339 | 54.339 |
| **Sensitivity** | 70.20% | 83.30% |
| **Specificity** | 68.10% | 66.20% |

**Abbreviations:** DPR, D-dimer to platelet ratio; msTBI, moderate-to-severe traumatic brain injury. The optimal cut-off value of **1.735** for log_10_-DPR was determined using the **Youden index** in the training cohort based on the Cox proportional hazards model. The same threshold was applied to the validation cohort to assess its diagnostic stability and generalizability. **Sensitivity** represents the ability of the threshold to correctly identify patients who died within 28 days. **Specificity** represents the ability of the threshold to correctly identify patients who survived for at least 28 days. **N** indicates the total number of patients included in each cohort.

**Supplementary Table 7. Sensitivity analysis of model performance using various DPR formulations and alternative predictive variables**

| **Model** | **C-index** | **AUC (28-day)** | **AIC** | **Number of predictors** |
| --- | --- | --- | --- | --- |
| Raw DPR (DPR-B model) | 0.815 | 0.852 | 530.26 | 3 |
| log_10_-DPR | 0.806 | 0.842 | 539.41 | 3 |
| Categorized DPR | 0.807 | 0.843 | 546.25 | 5 |
| D-dimer | 0.809 | 0.848 | 540.57 | 3 |
| log_10_-D-dimer | 0.804 | 0.842 | 544.22 | 3 |
| Platelet count (PLT) | 0.805 | 0.844 | 543.58 | 3 |
| D-dimer + PLT | 0.807 | 0.844 | 536.64 | 4 |
| Baseline Model | 0.759 | 0.800 | 560.29 | 1 |
| Full Model | 0.819 | 0.857 | 533.20 | 7 |

**Notes:** This table presents a sensitivity analysis comparing the raw DPR formulation (DPR-B model) against several alternative specifications. Unless otherwise specified, all models were adjusted for the baseline GCS and arterial blood gas (ABG) markers to maintain consistency with the primary model framework. Metrics (C-index, AUC, and AIC) are calculated based on the training cohort (n=239). The "Number of predictors" column indicates the count of independent variables included in each Cox proportional hazards model. The lower AIC value for the Raw DPR model compared to the Full Model suggests a more parsimonious model structure with comparable discriminative performance. **Abbreviations:** AUC, area under the receiver operating characteristic curve; AIC, Akaike information criterion; GCS, Glasgow Coma Scale; DPR, D-dimer-to-platelet ratio; PLT, platelet count.

**Supplementary Fig. 6. Comparative performance evaluation of predictive models.**

**Supplementary Fig. 6. Comparative performance evaluation of predictive models.** Comparison of C-index and 28-day time-dependent ROC curves across nine model specifications. All models were developed in the training cohort (n=239). The C-index comparison demonstrates the relative discriminative power of each formulation, while the ROC curves illustrate the sensitivity and specificity trade-offs for 28-day mortality prediction. **Abbreviations:** DPR, D-dimer-to-platelet ratio; PLT, platelet count.

**Supplementary Fig. 7. Calibration analysis of alternative predictive models in the training cohort.**

**Supplementary Fig. 7. Calibration analysis of alternative predictive models in the training cohort.** Calibration curves for sensitivity analysis models in the training cohort (n=239). These models replicate the configurations used in the validation cohort to provide a robust comparison of observed versus predicted mortality risk. (A) log_10_-DPR, (B) D-dimer, (C) PLT, (D) D-dimer plus PLT, (E) log_10_-D-dimer, and (F) Categorized DPR. The consistency between training and validation performance confirms the model robustness.

**Supplementary Fig. 8. Calibration analysis of alternative predictive models in the validation cohort.**

**Supplementary Fig. 8. Calibration analysis of alternative predictive models in the validation cohort.** Calibration curves for sensitivity analysis models in the validation cohort (n=99). The panels A–F correspond to alternative model specifications replacing the raw DPR variable: (A) log_10_-DPR, (B) D-dimer, (C) PLT, (D) D-dimer plus PLT, (E) log_10_-D-dimer, and (F) Categorized DPR. The solid line represents the actual performance of each model, while the diagonal dashed line denotes ideal calibration.

**Supplementary Table 8. Comparison of reclassification improvement (NRI and IDI) between the raw DPR model and alternative formulations**

| **Model Specification** | **Cohort** | **Metric** | **Estimate (95% CI)** | **P-value** |
| --- | --- | --- | --- | --- |
| **log_10_-DPR** | Train | NRI | 0.065 (-0.211, 0.359) | 0.624 |
|  |  | IDI | 0.002 (-0.020, 0.025) | 0.772 |
|  | Valid | NRI | -0.289 (-0.770, 0.164) | 1 |
|  |  | IDI | 0.008 (-0.022, 0.038) | 0.648 |
| **D-dimer** | Train | NRI | -0.378 (-0.677, -0.110) | 1 |
|  |  | IDI | -0.014 (-0.035, 0.003) | 1 |
|  | Valid | NRI | -0.520 (-0.955, -0.085) | 1 |
|  |  | IDI | -0.030 (-0.058, -0.007) | 1 |
| **PLT** | Train | NRI | -0.209 (-0.472, 0.063) | 1 |
|  |  | IDI | -0.024 (-0.064, 0.009) | 1 |
|  | Valid | NRI | -0.377 (-0.818, 0.052) | 1 |
|  |  | IDI | -0.043 (-0.102, 0.020) | 1 |
| **D-dimer + PLT** | Train | NRI | 0.155 (-0.096, 0.451) | 0.232 |
|  |  | IDI | 0.003 (-0.016, 0.020) | 0.66 |
|  | Valid | NRI | 0.252 (-0.129, 0.618) | 0.216 |
|  |  | IDI | 0.030 (0.009, 0.054) | <0.001 |
| **log_10_-D-dimer** | Train | NRI | -0.119 (-0.385, 0.140) | 1 |
|  |  | IDI | -0.015 (-0.043, 0.009) | 1 |
|  | Valid | NRI | -0.518 (-0.911, -0.084) | 1 |
|  |  | IDI | -0.042 (-0.078, -0.013) | 1 |
| **Categorized-DPR** | Train | NRI | 0.125 (-0.161, 0.415) | 0.384 |
|  |  | IDI | -0.008 (-0.033, 0.017) | 1 |
|  | Valid | NRI | 0.013 (-0.413, 0.465) | 1 |
|  |  | IDI | -0.012 (-0.047, 0.025) | 1 |

**Note:** All comparisons were performed against the raw DPR model as the reference. NRI and IDI were calculated using bootstrapping (R=500). P-values < 0.05 are generally considered statistically significant. **Abbreviations:** NRI, Net Reclassification Improvement; IDI, Integrated Discrimination Improvement; CI, Confidence Interval.

**Supplementary Table 9. Rates of missing follow-up CT within 72 hours**

| **Subgroup** | **Total Cohort (n=340)** | **Training Set (n=239)** | **Validation Set (n=101)** |
| --- | --- | --- | --- |
| **No follow-up CT (overall)** | 9.41% | 10.04% | 7.92% |
| **Early death (≤72h)** | 54.05% | 53.85% | 54.55% |
| **Late death (>72h)** | 22.73% | 25.81% | 15.38% |
| **Survivor** | 0.77% | 1.10% | 0% |
| **Death** | 37.04% | 38.60% | 33.33% |
| **P (Early death vs Late death)** | 0.004 | 0.0303 | 0.0426 |
| **P (Survivor vs Death)** | <0.001 | <0.001 | <0.001 |

**NOTE:** Early death was defined as death within 72 hours of admission; late death was defined as death after 72 hours but within 28 days; survivors were patients who survived beyond 28 days; death included both early and late deaths. **Abbreviations:** CT, computed tomography; h, hours.

**Supplementary Table 10. Univariate and multivariable logistic regression analysis of predictors for early neurological deterioration (END) in the training cohort.**

| **Variable** | **Univariate OR (95% CI)** | **Univariate *P*-value** | **Multivariable OR (95% CI)** | **Multivariable *P*-value** |
| --- | --- | --- | --- | --- |
| **GCS** | 0.780 (0.684–0.889) | 0.0002 | 0.853 (0.740–0.984) | 0.029 |
| **ABG** | 1.204 (1.104–1.313) | <0.001 | 1.127 (1.022–1.243) | 0.017 |
| **DPR (per 1 SD)** | 3.218 (2.001–5.173) | <0.001 | 2.223 (1.367–3.614) | 0.001 |
| **Age** | 1.028 (1.007–1.049) | 0.008 | 1.023 (0.999–1.048) | 0.059 |
| **Temperature** | 0.515 (0.314–0.847) | 0.009 | 0.515 (0.303–0.875) | 0.014 |
| **INR** | 2.394 (0.794–7.217) | 0.121 | — | — |
| **MAP** | 0.991 (0.976–1.005) | 0.205 | — | — |
| **Sodium** | 0.981 (0.914–1.053) | 0.596 | — | — |
| **D-Dimer (per 1 SD)** | 3.284 (2.122–5.085) | <0.001 | — | — |
| **Creatinine** | 1.001 (0.992–1.009) | 0.905 | — | — |

**NOTE:** Variables with P < 0.05 in univariate analysis were entered into the multivariable model. Multivariable logistic regression was adjusted for temperature, GCS, ABG, DPR, and age. **Abbreviations:** OR, odds ratio; CI, confidence interval; GCS, Glasgow Coma Scale; ABG, admission blood glucose; DPR, D-dimer to platelet ratio; MAP, mean arterial pressure; INR, international normalized ratio.

**Supplementary Fig. 9. Receiver operating characteristic (ROC) curve of DPR for predicting early neurological deterioration (END) in the training cohort.** The area under the curve (AUC) was 0.724 (95% confidence interval: 0.643–0.806), indicating moderate discriminative ability for identifying patients at risk of END within 72 hours of admission.

**Supplementary Fig. 10. Receiver operating characteristic (ROC) curve of DPR for predicting early neurological deterioration (END) in the validation cohort.** The area under the curve (AUC) was 0.874 (95% confidence interval: 0.786--0.962), indicating good discriminative ability for identifying patients at risk of END within 72 hours of admission.

**Supplementary Table 11. Sensitivity analysis of factors associated with END in the training cohort (n=206).**

| **Variable** | **Univariate OR (95% CI)** | **Univariate P-value** | **Multivariable OR (95% CI)** | **Multivariable P-value** |
| --- | --- | --- | --- | --- |
| **GCS** | 0.859 (0.748–0.987) | 0.032 | 0.914 (0.787–1.060) | 0.233 |
| **ABG** | 1.173 (1.060–1.298) | 0.002 | 1.110 (0.983–1.252) | 0.092 |
| **DPR (per 1 SD)** | 2.630 (1.517–4.559) | <0.001 | 1.869 (1.049–3.329) | 0.034 |
| **Age** | 1.026 (1.002–1.051) | 0.034 | 1.020 (0.993–1.047) | 0.151 |
| **Temperature** | 0.516 (0.275–0.967) | 0.039 | 0.554 (0.290–1.059) | 0.074 |
| **INR** | 1.776 (0.448–7.041) | 0.414 | — | — |
| **MAP** | 1.000 (0.981–1.019) | 0.998 | — | — |
| **Sodium** | 0.923 (0.838–1.016) | 0.1 | — | — |
| **D-Dimer (per 1 SD)** | 2.750 (1.645–4.597) | <0.001 | — | — |
| **Creatinine** | 0.998 (0.986–1.009) | 0.686 | — | — |

**Abbreviations:** OR, odds ratio; CI, confidence interval; SD, standard deviation; GCS, Glasgow Coma Scale; ABG, Admission Blood Glucose; DPR, D-dimer to Platelet Ratio; INR, International Normalized Ratio; MAP, Mean Arterial Pressure.
